# Post-earthquake urban reconstruction, land use, and tropospheric NO₂ exposure in Malatya, Türkiye

**DOI:** 10.3389/fpubh.2026.1934899

**Published:** 2026-09-14

**Authors:** Enes Karadeniz

**Affiliations:** Department of Geography, Faculty of Arts and Science, Inonu University, Malatya, Türkiye

**Keywords:** environmental exposure, land use, NO_2_, public health, Sentinel-5P, XGBoost

## Abstract

Land-use decisions made during post-disaster reconstruction can reshape transport systems, built-up environments, and the spatial distribution of environmental exposures. However, these relationships remain insufficiently examined in earthquake-affected cities. This study evaluates the temporal and spatial patterns of tropospheric nitrogen dioxide (NO₂) in Malatya, Türkiye, during 2019–2025 and compares the 2019–2022 baseline with the 2023–2025 reconstruction period. Sentinel-5P TROPOMI observations were integrated with land use/land cover, road density, elevation, NDVI, and NDBI within a common spatial framework comprising 108,626 pixel-year observations. Tropospheric NO₂ was modelled using XGBoost, variable contributions were interpreted through SHAP, and spatial dependence was assessed using Global Moran’s *I*. High NO₂ levels were consistently concentrated along the Battalgazi-Yeşilyurt urban axis, where dense road networks, artificial surfaces, and intensive urban functions overlap, whereas lower values were observed in higher-elevation and lower-density areas. Land use/land cover had the greatest gain-based contribution to the model, while elevation exerted a stronger influence on pixel-level predictions. The model showed moderate predictive performance on the holdout test set (*R*^2^ = 0.5941, RMSE = 2.816 μmol m^−2^, and MAE = 1.865 μmol m^−2^). Annual Global Moran’s *I* values ranged from 0.9893 to 0.9922 (*p* = 0.001), indicating strong spatial clustering of tropospheric NO₂ throughout the study period. The reconstruction period was characterized by a more fragmented and spatially heterogeneous NO₂ pattern rather than a uniform province-wide increase. These findings suggest that changing transport flows, built-up development, topographic controls, and post-disaster spatial reorganization may jointly differentiate environmental exposure conditions. The study provides interpretable spatial evidence for health-sensitive land-use planning, air-quality monitoring, and post-disaster urban management.

## 1 Introduction

Land use decisions are among the fundamental planning components that directly shape the environmental conditions to which urban populations are exposed. The spatial organization of cities determines where traffic intensity is concentrated, how built-up surfaces expand, at what scale and in which locations green spaces are distributed, and how local ventilation conditions affect pollutant accumulation. Therefore, land use should be considered not only as a matter of spatial regulation or physical planning, but also as a public health component that determines the distribution of environmental exposure. Rapid urbanization processes, the expansion of transport infrastructure, and the increase in built-up surfaces generate multidimensional effects, particularly on traffic-related emissions, heat stress, noise, access to green spaces, and air quality (1–5). In this context, understanding how urban land use patterns produce environmental exposure conditions and through which spatial factors they intensify is critically important for health-sensitive urban planning.

Among urban air pollutants, nitrogen dioxide (NO₂) is an important indicator for evaluating the relationship between land use and public health, as it represents transport- and combustion-related emissions. NO₂ is strongly associated with motor vehicle traffic, industrial activities, energy consumption, and other fossil fuel combustion processes. Epidemiological studies have shown that short- and long-term exposure to NO₂ is associated with respiratory and cardiovascular health outcomes (5–7). However, this study does not directly measure disease, mortality, hospital admissions, or individual health outcomes. Therefore, NO₂ is treated here not as a direct health outcome, but as an atmospheric indicator representing environmentally meaningful exposure conditions from a public health perspective. The relatively short atmospheric lifetime of NO₂ and its strong spatial gradients near emission sources make it a suitable variable for evaluating exposure patterns associated with land use, transport intensity, and urban morphology at local and regional scales (8–10).

The spatial distribution of NO₂ cannot be explained solely by emission quantities. Land use, the transport network, vegetation, built-up surfaces, and topographic conditions jointly influence this distribution. While built-up areas and high road density represent spatial units where traffic-related emissions and intra-urban human activities are concentrated, natural and semi-natural surfaces, vegetation density, and open areas can create different dispersion and accumulation conditions. Land use regression and GIS-based air pollution modelling studies have shown that road length, road type, population density, land cover classes, and physical geography variables play an important role in explaining the spatial variation of NO₂ and other air pollutants (9, 11, 12). The effect of vegetation on air quality is context-dependent. While vegetation can reduce pollutant accumulation under some conditions, it has also been noted that dense vegetation structures that restrict airflow, particularly at the street scale, may weaken pollutant dispersion (13, 14). Topography, particularly in basin and valley systems, can restrict air circulation and cause pollutants to accumulate in certain areas (15). Therefore, the assessment of NO₂ distribution requires an integrated spatial approach that includes not only emission sources but also surface characteristics and physical geography conditions.

Monitoring such environmental exposure environments requires analytical methods capable of jointly evaluating spatially continuous observations and different data sources. Although ground-based air quality stations provide highly accurate measurements, they may remain limited in sufficiently representing urban and regional pollutant gradients in areas where station networks are sparse. Satellite-based observations such as Sentinel-5P TROPOMI partly overcome this limitation by enabling tropospheric NO₂ to be monitored comparably over large areas (10, 16, 17). When these observations are combined with GIS-based indicators of land use, road networks, topography, vegetation, and built-up areas, they allow the spatial determinants of air pollution patterns to be evaluated in greater detail (8). In recent years, tree-based machine learning algorithms such as XGBoost have been increasingly used in air pollution prediction studies because they can model nonlinear relationships and threshold effects among environmental variables (18, 19). In addition, explainable artificial intelligence approaches such as SHAP quantitatively reveal the contribution of each variable to model predictions, making machine learning outputs more interpretable in the context of planning and environmental health (19, 20).

Malatya Province provides a distinctive study area for evaluating the relationship between land use, air quality, and post-disaster reconstruction. The 6 February 2023 earthquakes had serious impacts on the built environment across the province and initiated a new process in the post-earthquake period in terms of reconstruction, temporary settlement, transport mobility, and the spatial organization of urban functions. Such large-scale disasters and subsequent reconstruction periods can reshape environmental exposure conditions by altering transport flows, heavy vehicle mobility, construction activities, temporary settlement areas, and land use patterns. Indeed, studies on post-disaster environmental conditions have shown that earthquakes and reconstruction processes can create local and temporal pressures on air quality (21, 22). These effects do not emerge in the same way in every region. Local topography, the spatial direction of reconstruction, the organization of the transport network, and the existing land use structure determine the environmental consequences of this process. Therefore, Malatya constitutes a meaningful example for assessing how post-disaster spatial reorganization is associated with NO₂ patterns.

Satellite-based NO₂ monitoring is increasingly combined with machine learning to estimate surface concentrations and examine their spatial drivers (23–25). Recent studies have integrated TROPOMI observations with land use, topography, population, meteorological, and traffic-related variables using tree-based models and SHAP. Examples include XGBoost-based NO₂ mapping across Europe (26), ground-level estimation across Southeastern Europe (27), comparisons of different tree-based algorithms using geostationary and polar-orbiting satellite observations (28), and Random Forest–SHAP analysis of NO₂ patterns across 346 Chinese cities (29). TROPOMI-based land-use regression has also demonstrated the importance of satellite observations and road-network variables in estimating surface NO₂ concentrations (30). In Türkiye, related applications include NO₂ forecasting and population-exposure assessment in the Marmara Region (31), ground-level NO₂ estimation in Istanbul (32), explainable assessments of traffic and meteorological influences (33), and CatBoost–SHAP-based air-quality forecasting in Başakşehir (34). Post-earthquake research using TROPOMI has additionally documented short-term changes in NO₂ levels across provinces affected by the 6 February 2023 earthquakes (35).

However, studies combining land-use characteristics, topographic controls, satellite-derived NO₂ observations, and interpretable machine learning within a post-earthquake reconstruction context remain limited. Research in Türkiye has focused mainly on large metropolitan areas and ground-station-based estimation, while existing post-earthquake studies have largely examined short-term changes rather than multi-year spatial patterns during reconstruction. This gap is particularly important for public health-oriented land use planning. This is because site selection, transport, housing, and infrastructure decisions made during post-disaster reconstruction periods can have lasting effects on long-term exposure patterns. This study contributes to this gap in three ways. First, it evaluates the relationship between land use and public health not directly through epidemiological health outcomes, but through NO₂-based environmental exposure conditions. Second, by comparing the pre-earthquake period of 2019–2022 and the post-earthquake period of 2023–2025, it examines the relationship between post-disaster spatial reorganization and tropospheric NO₂ patterns. Third, by integrating Sentinel-5P TROPOMI observations with GIS-based environmental variables, XGBoost, and SHAP, it enables an interpretable assessment of the land use, topographic, and anthropogenic factors that shape NO₂ distribution.

The aim of this study is to evaluate the temporal and spatial distribution of tropospheric NO₂ in Malatya Province during the 2019–2025 period and to explain the relationship between this distribution and land use, topographic structure, and anthropogenic indicators through an XGBoost- and SHAP-based modelling approach. By comparing the pre-earthquake period (2019–2022) with the post-earthquake period (2023–2025), the study aims to reveal how changing urban and environmental conditions during the post-disaster reconstruction process are associated with NO₂ patterns. In this respect, the research establishes a methodological and conceptual bridge between land use planning, environmental health assessment, and post-disaster urban management, producing exposure-oriented spatial evidence for public-health-sensitive planning decisions.

## 2 Materials and methods

### 2.1 Study area

This study was conducted within the boundaries of Malatya Province, located in the Upper Euphrates Section of Türkiye’s Eastern Anatolia Region. The province is situated between valley systems shaped by the Euphrates River and its tributaries and high topographic units bordered by Yama Mountain in the north and the Malatya Mountains in the south (Figure 1). This morphological structure causes marked elevation differences over short distances and spatial variability in microclimatic conditions (36, 37). In particular, plain floors, valley corridors, and semi-enclosed areas surrounded by high topographic thresholds can influence the transport, dilution, and accumulation of pollutants under atmospheric stagnation and thermal inversion conditions.

**Figure 1:**
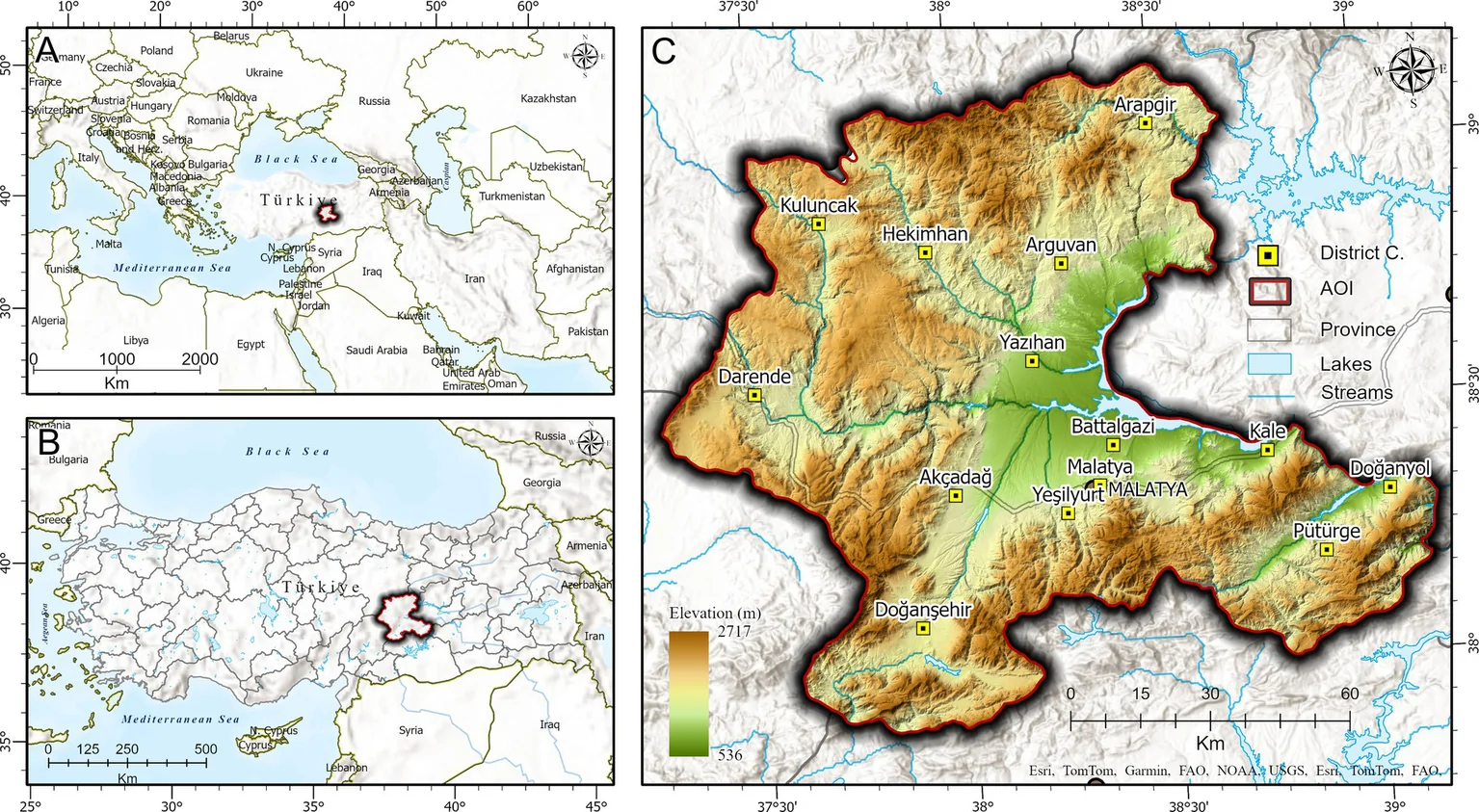
Location and geographical setting of the study area: **(A)** The location of Türkiye within the surrounding region, **(B)** The location of Malatya Province within Türkiye, and **(C)** The topographic setting and administrative districts of Malatya Province.

Malatya Province provides a suitable study area for evaluating tropospheric NO₂ distribution together with topographic, urban, and transport-related determinants. The districts of Battalgazi and Yeşilyurt represent the areas where population density, transport networks, commercial activities, and urban expansion pressure are most pronounced. In contrast, anthropogenic pressure is relatively limited in the higher-elevation and lower-density districts in the north and northwest of the province. This spatial contrast requires NO₂ distribution to be assessed not only in relation to the location of emission sources, but also together with topographic control, land use, and atmospheric dispersion conditions. The importance of the study area has further increased with the urban reconstruction process that emerged after the 6 February 2023 earthquakes. The development of new settlement areas, changes in transport mobility, and the intensification of reconstruction activities have created a new spatial context that may affect human-induced emission dynamics, particularly in the city center and its surroundings. Therefore, the study design covering the 2019–2025 period enables pre- and post-earthquake tropospheric NO₂ patterns to be evaluated together with environmental and anthropogenic determinants.

### 2.2 Study design and datasets

In this study, an integrated research design based on remote sensing, geographic information systems, and machine learning was adopted to evaluate the temporal, seasonal, and spatial patterns of tropospheric NO₂ distribution in Malatya Province. The dependent variable of the study was the tropospheric NO₂ column density obtained from the Sentinel-5P TROPOMI sensor. Sentinel-5P data are widely used in air quality studies because they provide usable results for monitoring atmospheric pollutants in Türkiye and at different regional scales (38, 39). The explanatory variables were composed of topographic, surface, and anthropogenic indicators that may affect NO₂ distribution. In this context, elevation, slope, and aspect variables were derived from the ALOS PALSAR digital elevation model; NDVI and NDBI indices were calculated from Sentinel-2 imagery; distance to roads and road density indicators were produced from OpenStreetMap road data; TÜİK population data were used to represent anthropogenic pressure; and ESRI 10 m Land Cover data were included in the model to represent the land use/land cover structure. The datasets used in the study and their analytical roles are presented in Table 1.

**Table 1:** Datasets used in the study and their analytical roles.

Dataset	Source	Period/reference year	Original spatial resolution: TROPOMI native ground-pixel footprint	Analysis resolution	Derived variable/analytical role
Sentinel-5P TROPOMI NO₂	Google Earth Engine, COPERNICUS/S5P/OFFL/L3_NO2	2019–2025	~5.5 km × 3.5 km (TROPOMI ground-pixel footprint)	1,000 m analytical grid	Dependent variable; annual, seasonal, pre-earthquake and post-earthquake NO₂ surfaces
ALOS PALSAR DEM	ALOS PALSAR	Static topographic layer	12.5 m	1,000 m	Elevation, slope and aspect
Sentinel-2 imagery	Copernicus Sentinel-2	Study-period representative imagery	10 m	1,000 m	NDVI and NDBI indices
Road network	OpenStreetMap	Current road network	Vector	1,000 m	Distance to roads and road density
Population data	TÜİK	Closest available population year	Administrative/statistical unit	1,000 m	Population density as an anthropogenic pressure indicator
Land use/land cover	ESRI land cover	Study-period representative LULC layer	10 m	1,000 m	LULC classes representing surface characteristics

The datasets integrated in this study differed in their native temporal resolution; therefore, a temporal harmonization procedure was applied before constructing the machine-learning dataset. The analysis was organized around annual temporal units covering the 2019–2025 period, while two broader periods were additionally defined for the earthquake-related comparison: the pre-earthquake period (2019–2022) and the post-earthquake period (2023–2025). This framework enabled atmospheric observations and surface-based explanatory variables to be integrated at a common temporal scale while preserving the distinction between conditions before and after the February 2023 earthquakes.

Sentinel-5P TROPOMI constituted the atmospheric target dataset. Daily Level-3 tropospheric NO₂ observations (COPERNICUS/S5P/OFFL/L3_NO2) were obtained and processed in Google Earth Engine. Following quality filtering, valid daily observations were aggregated into monthly composite surfaces, and these monthly products were subsequently aggregated to derive annual tropospheric NO₂ surfaces for each year from 2019 to 2025. The annual NO₂ surfaces were used as the dependent variable in the XGBoost framework, whereas the monthly observations were retained for temporal and seasonal analyses. Therefore, the XGBoost output represents Sentinel-5P-derived tropospheric NO₂ column density rather than ground-level NO₂ concentration.

Annual NDVI and NDBI layers were derived from Sentinel-2 Level-2A imagery acquired in June of each year from 2019 to 2025. These layers and the annual ESRI LULC data were paired with the Sentinel-5P NO₂ surface for the corresponding year. Elevation derived from the ALOS PALSAR DEM was treated as a static topographic predictor, while road density represented transport-network intensity. All predictor layers were harmonized to the common 1,000 m analytical grid. The final predictor set included elevation, LULC, road density, NDVI, NDBI, and population density. Population density was retained as a predictor but produced zero gain and zero SHAP contribution.

Annual pixel-year observations were grouped into the pre-earthquake (2019–2022) and post-earthquake (2023–2025) periods for comparative analysis. Period membership was not included as a predictor, and separate earthquake-specific models were not fitted. Meteorological variables were not included in the predictor set.

The research workflow was carried out in four main stages. In the first stage, Sentinel-5P TROPOMI NO₂ data for the 2019–2025 period were obtained in the Google Earth Engine environment, quality control steps were applied, and NO₂ surfaces suitable for annual, seasonal, pre-earthquake, and post-earthquake analyses were produced. In the second stage, topographic, surface, and anthropogenic variables were prepared in a GIS environment, and all layers were arranged according to the Malatya provincial boundary. In the third stage, the dependent and independent variables were combined within a common pixel structure to create a spatial data matrix suitable for modelling. In the final stage, a tropospheric NO₂ prediction model was developed using the XGBoost algorithm, model performance was evaluated with error metrics, and variable contributions were interpreted using the SHAP approach (18, 40). The overall methodological workflow of the study is presented in Figure 2.

**Figure 2:**
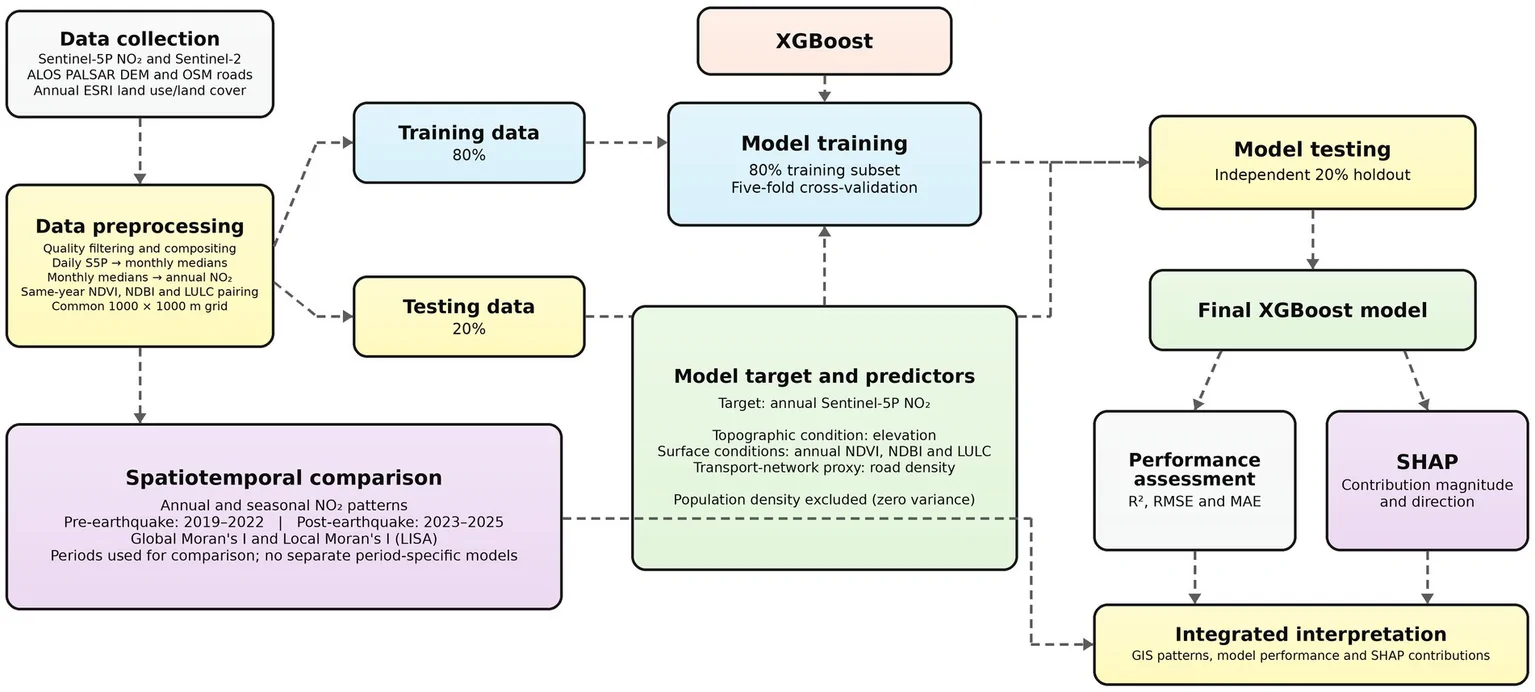
Overall methodological workflow of the study.

In order to enable the joint analysis of datasets with different resolutions, formats, and sources, all layers were harmonized into a common spatial analysis framework before modelling. In this context, raster- and vector-based data were clipped to the Malatya provincial boundary, arranged in a common coordinate system, and transferred to a 1,000 × 1,000 m analysis grid suitable for pixel-based comparison. This procedure was applied not as a resolution enhancement step that increases the original observational capacity of the Sentinel-5P product, but as a harmonization process that allows different data sources to be compared through the same spatial unit.

### 2.3 Sentinel-5P TROPOMI NO₂ data and pre-processing procedure

The dependent variable representing tropospheric NO₂ distribution was obtained from the TROPOMI sensor aboard the Sentinel-5 Precursor satellite operated under the Copernicus Programme. TROPOMI is a wide-coverage sensor used to monitor atmospheric trace gases and allows tropospheric NO₂ column density to be evaluated at the regional scale (41–43). In this study, the COPERNICUS/S5P/OFFL/L3_NO2 product available in the Google Earth Engine data catalogue was used, and observations for the 2019–2025 period were analysed. The use of tropospheric column NO₂ data is based on its ability to monitor pollutant patterns in a comparable way in areas where ground-level measurement networks are limited in terms of spatial continuity (41, 44).

The data pre-processing procedure was carried out in three main stages. In the first stage, only pixels satisfying the condition qa_value > 0.75 were included in the analysis to exclude low-reliability observations. This quality filter helps reduce low-reliability observations that may result from cloud effects, snow cover, measurement geometry, and atmospheric conditions. In the second stage, daily NO₂ observations were converted into monthly median composites to limit the effects of short-term atmospheric fluctuations and outliers. The median approach was preferred because it reduces the influence of short-term anomalies on temporal averages, particularly in satellite-based atmospheric datasets. In the third stage, annual NO₂ surfaces for the 2019–2025 period were produced from the monthly composites. In addition, multi-year seasonal composites representing spring, summer, autumn, and winter were generated for seasonal comparisons. To evaluate the earthquake effect at the period level, 2019–2022 was defined as the pre-earthquake period and 2023–2025 as the post-earthquake period, and average NO₂ surfaces were calculated for both periods. All resulting NO₂ rasters were clipped to the Malatya provincial boundary, transferred to the common analysis grid, and prepared in the modelling environment so that they could be matched at the pixel level with GIS-based explanatory variables.

### 2.4 Generation of GIS-based independent variables

The independent variables used to explain tropospheric NO₂ distribution were prepared in a GIS environment to represent topographic structure, surface characteristics, and anthropogenic pressure conditions. Topographic variables were derived from the ALOS PALSAR digital elevation model. In this context, elevation, slope, and aspect layers were produced, and the possible effects of terrain morphology on the transport, dilution, and accumulation of pollutants were included in the model dataset. To represent surface characteristics, NDVI and NDBI indices were calculated from Sentinel-2 imagery. NDVI was used as an explanatory variable representing vegetation density, while NDBI was used to represent the spatial distribution of built-up surfaces. Variables representing anthropogenic pressure were produced from the OpenStreetMap road network and TÜİK population data. Using OSM road data, distance to roads and road density layers were generated. The distance-to-road variable was calculated based on the Euclidean distance of each raster cell to the nearest road network element:


$$D_{i}=\min_{r\in R}d(p_{i},r)$$ (1)

Here, $D_{i}\;$ denotes the distance of raster cell i to the nearest road network element; $p_{i}$ denotes the center point of the raster cell; R denotes the set of road network elements; and $d(p_{i},r)\;$ denotes the Euclidean distance between the cell center and the nearest location on the road network. The road density layer was used to represent the spatial intensity of the transport network, while the population density layer was used to represent the distribution of human activities and urban pressure. The land use/land cover variable was obtained from the ESRI 10 m Land Cover dataset. This layer was incorporated into the model to represent the possible effects of artificial surfaces, agricultural areas, natural and semi-natural surfaces, and other land cover classes on NO₂ distribution. Since LULC is a categorical variable, it was not normalized like the continuous variables; instead, its class information was preserved and included in the model dataset. Min–Max normalization was applied to the continuous explanatory variables to reduce scale effects arising from different measurement units:


$$X_{i}^{\prime }=\frac{X_{i}-X_{\min}}{X_{\max}-X_{\min}}$$ (2)

Here, $X_{i}\;$ denotes the original value in the corresponding raster cell; $X_{\min}$ and $X_{\max}\;$ denote the minimum and maximum values of the relevant variable within the study area; and $X_{i}^{\prime }$ represents the normalized value. This procedure was applied only to continuous variables, while the class structure of the categorical LULC variable was preserved.

### 2.5 Machine learning algorithm: XGBoost modelling approach

XGBoost, a tree-based ensemble learning algorithm based on the gradient boosting principle, was used for the pixel-based prediction of tropospheric NO₂ concentrations. XGBoost is a modelling approach that reduces previous prediction errors through sequential decision trees, limits the risk of overfitting through regularization, and flexibly represents nonlinear relationships among variables (18). In this study, the modelling process was carried out in the Python environment using the XGBRegressor class.

The XGBoost model was developed to estimate annual Sentinel-5P-derived tropospheric NO₂ column density using elevation, LULC, road density, NDVI, and NDBI as predictors. The model was constructed from pixel-year observations covering 2019–2025 and was intended to examine potentially nonlinear relationships between the selected predictors and the satellite-derived NO₂ target. It was not designed to forecast NO₂ for future years or to estimate ground-level NO₂ concentrations.

Following spatial harmonization to the common 1,000 m grid, the modelling dataset was divided using an 80/20 random holdout split. Eighty percent of the observations were used for model training, while the remaining 20% formed the held-out test subset. Five-fold cross-validation was applied within the training subset during model development. Because spatial and temporal blocking was not used, neighbouring pixels and observations from the same spatial units in different years could occur in both subsets. The test results therefore represent held-out performance under the adopted random split rather than fully independent spatial or temporal transferability.

Tropospheric NO₂ column-density values obtained from Sentinel-5P TROPOMI were converted from mol m^−2^ to μmol m^−2^. After NoData cells were removed, the final dataset comprised 108,626 pixel-year observations. The general prediction structure of the XGBoost model is expressed as follows:


$${\hat{y}}_{i}=\sum_{k=1}^{K}f_{k}(x_{i}),f_{k}\in ℱ$$ (3)

Here, ${\hat{y}}_{i}\;$ denotes the predicted tropospheric NO₂ value for raster cell i ; $x_{i}\;$ denotes the vector of explanatory variables for the corresponding cell; $f_{k}\;$ denotes the k -th decision tree; K denotes the total number of decision trees in the model; and ℱ denotes the space of regression trees. The model formed the final prediction function by adding a new decision tree at each iteration to reduce previous prediction errors.

### 2.6 SHAP-based model interpretability and variable contribution analysis

The SHAP approach was used to improve the interpretability of the predictions obtained from the XGBoost model and to assess the contribution of each explanatory variable to tropospheric NO₂ predictions. SHAP is an explainable artificial intelligence method based on Shapley values from game theory, and it calculates the marginal contribution of each variable to the model output at the observation level (20, 45). This approach allows not only the general importance of variables in tree-based models to be evaluated, but also the increasing or decreasing effect of variables on the prediction at each pixel. Therefore, SHAP analysis was used to reduce the black-box nature of the model and to interpret more transparently the environmental control mechanisms shaping NO₂ distribution. In the SHAP approach, each model prediction is expressed as the sum of the base prediction value and the variable contributions:


$$\hat{f}(x_{i})=\phi_{0}+\sum_{j=1}^{M}\phi_{j}$$ (4)

Here, $\hat{f}(x_{i})\;$ denotes the tropospheric NO₂ value predicted by the model for observation i ; $\phi_{0}$ denotes the model’s average base prediction value; $\phi_{j}\;$ denotes the contribution of the j -th explanatory variable to the relevant prediction; and M denotes the total number of variables. Positive SHAP values indicate that the relevant variable contributes to increasing the predicted NO₂ value at a given pixel, whereas negative SHAP values indicate a decreasing effect on the prediction. Variable contributions were evaluated at two levels. First, the overall contribution level of the variables to the model predictions was determined using mean absolute SHAP values. This stage provided a complementary interpretation basis to the gain-based variable importance results of XGBoost. Second, through SHAP summary plots, the direction and magnitude of the effect of each variable on the prediction across its value range were examined. Thus, not only the contribution level of variables such as elevation, road density, NDVI, NDBI, and land use/land cover to the model, but also the conditions under which they increased or decreased NO₂ predictions, were evaluated.

### 2.7 Evaluation of model performance

The predictive performance of the XGBoost model was evaluated using holdout test outputs and cross-validation results. The coefficient of determination ( $R^{2}$ ), root mean square error (RMSE), and mean absolute error (MAE) were used to measure model performance. These metrics were calculated to evaluate the explanatory power of the model, its sensitivity to large errors, and the average prediction deviation, respectively.


$$R^{2}=1-\frac{\sum_{i=1}^{n}{\left(y_{i}-{\hat{y}}_{i}\right)}^{2}}{\sum_{i=1}^{n}{\left(y_{i}-\overset{ˉ}{y}\right)}^{2}}$$ (5)

$$\text{RMSE}=\sqrt{\frac{1}{n}\sum_{i=1}^{n}{\left(y_{i}-{\hat{y}}_{i}\right)}^{2}}$$ (6)

$$\mathrm{MAE}=\frac{1}{n}\sum_{i=1}^{n}|y_{i}-{\hat{y}}_{i}|$$ (7)

Here, $y_{i}\;$ denotes the observed tropospheric NO₂ value; ${\hat{y}}_{i}\;$ denotes the NO₂ value predicted by the model; $\overset{ˉ}{y\;}$ denotes the mean of the observed values; and n denotes the total number of observations.

Collectively, Equations 1–7 define the spatial predictors, data transformation, model structure, SHAP decomposition, and model-performance metrics used in the analysis.

As a supporting analysis for the spatial consistency of model predictions, spatial dependence in the tropospheric NO₂ distribution was evaluated using the Global Moran’s *I* statistic. This analysis was applied to determine whether high or low NO₂ values were spatially randomly distributed or whether they tended to cluster in specific areas. In addition, anomaly assessments were performed on monthly and annual NO₂ series to examine temporal deviations during the 2019–2025 period. These additional controls contributed to the interpretation of the model results not only in terms of predictive performance, but also in terms of the consistency of spatial and temporal patterns.

## 3 Results

### 3.1 Temporal and spatial variation of tropospheric NO₂ concentrations (2019–2025)

Sentinel-5P TROPOMI data for the 2019–2025 period reveal that the distribution of tropospheric NO₂ in the atmosphere of Malatya Province exhibited marked temporal fluctuations and strong spatial differentiation. Annual raster analyses show that NO₂ concentrations changed considerably throughout the study period in terms of both intensity and spatial organization (Figure 3). The findings indicate that the atmospheric NO₂ system cannot be explained solely by the intensity of emission sources, but is controlled by a multi-component environmental system shaped jointly by topographic structure, atmospheric circulation conditions, transport intensity, urbanization processes, and anthropogenic activities.

**Figure 3:**
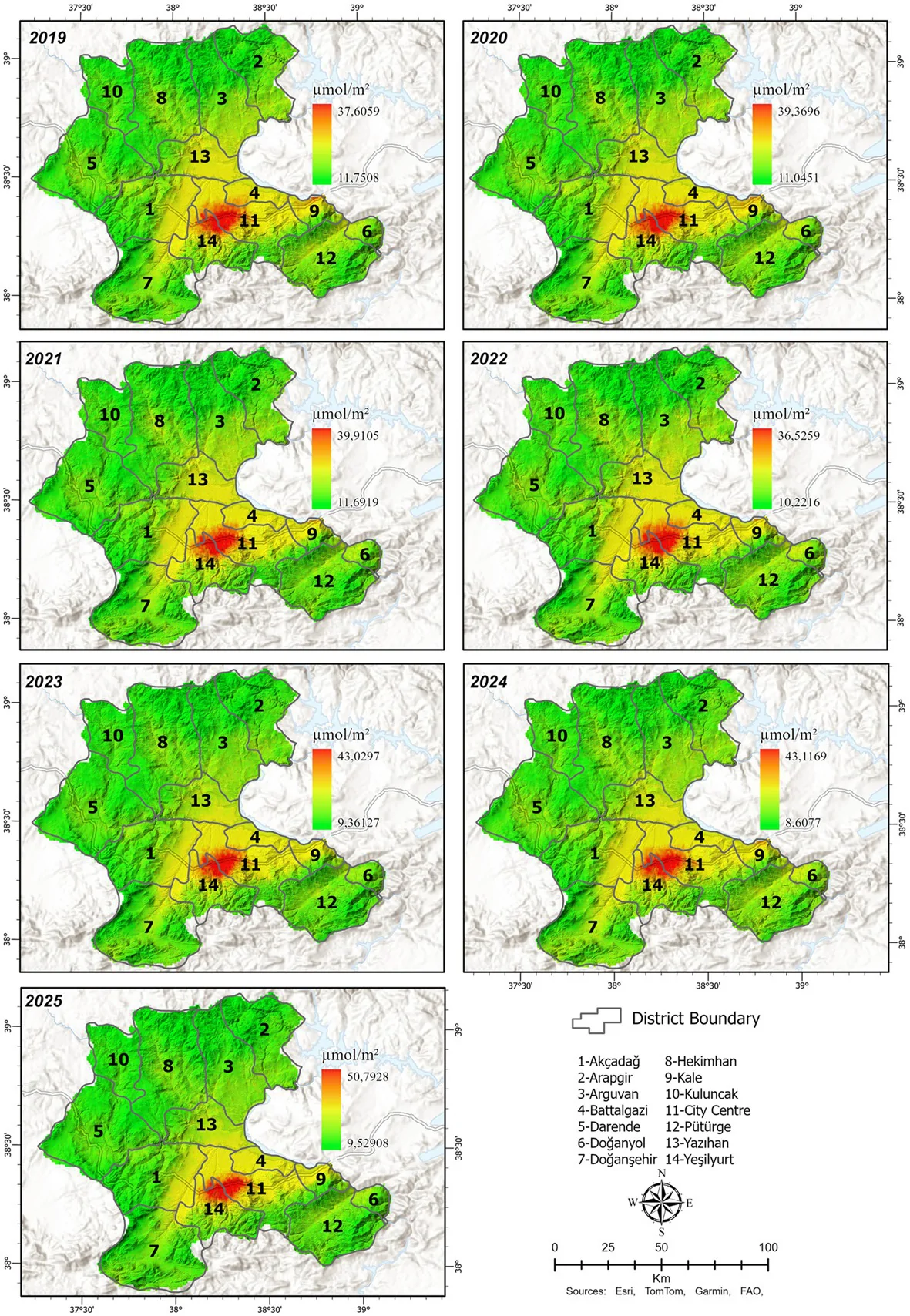
Temporal and spatial variation of NO_2_ in Malatya Province during the 2019–2025 period.

During the study period, annual minimum NO₂ values ranged approximately between 9.55 and 11.69 μmol m^−2^, while maximum values ranged between 38.08 and 46.31 μmol m^−2^. Although mean annual concentrations varied within a relatively narrow range of approximately 17.87–18.34 μmol m^−2^, the overall increase in maximum values and standard deviation toward the end of the study period indicates that the spatial distribution became more heterogeneous. In particular, the increase in standard deviation from approximately 3.28 μmol m^−2^ in 2019 to 3.99 μmol m^−2^ in 2025 indicates greater spatial dispersion and a stronger contrast between lower- and higher-concentration areas. When annual mean concentrations are examined, the mean NO₂ value was approximately 18.01 μmol m^−2^ in 2019 and remained nearly stable at 17.97 μmol m^−2^ in 2020. Although the change in the annual mean was limited, reduced transport mobility, economic activity, and intra-urban movement during the COVID-19 pandemic may have influenced the NO₂ distribution observed within 2020. This possibility is consistent with studies examining spatial–temporal changes in NO₂ and air quality in Türkiye during the COVID-19 period using Sentinel-5P and GEE-based analyses (46, 47). The annual mean subsequently increased to approximately 18.11 μmol m^−2^ in 2021, coinciding with the re-intensification of anthropogenic activities.

Although annual mean values remained relatively stable in 2022 and 2023, comparatively high maximum values continued to occur. In 2023, the mean NO₂ value was approximately 17.87 μmol m^−2^, whereas the maximum reached 40.82 μmol m^−2^, indicating the presence of localized high-concentration areas despite the relatively stable province-wide mean. Because meteorological variables were not included in the analysis, the processes underlying these localized maxima cannot be attributed to specific atmospheric conditions. The most notable change in the study period was observed toward 2024 and 2025. In particular, the maximum NO₂ value reached approximately 46.31 μmol m^−2^ in 2025, representing the highest value recorded during the study period. The increase in standard deviation during the same period indicates greater spatial heterogeneity in the NO₂ distribution. The widening difference between mean and maximum values shows that localized high-concentration areas became more pronounced rather than indicating a uniform province-wide increase.

When the spatial distribution maps are examined, NO₂ concentrations are seen to be concentrated particularly in the southern and southeastern parts of Malatya. In these areas, annual mean concentrations reached approximately 26–34 μmol m^−2^. In contrast, NO₂ levels remained within approximately 15–22 μmol m^−2^ in the high topographic areas in the north and northwest of the province. This indicates the presence of a strong north–south spatial gradient reaching approximately 10–12 μmol m^−2^ across the study area. The most prominent spatial pattern is observed in the districts of Battalgazi and Yeşilyurt. These districts represent the areas with the highest population density, dense transport networks, commercial activities, and built-up pressure. In Battalgazi district in particular, continuous high-density zones occur due to the historical urban center and intensive commercial activities. In Yeşilyurt district, the recent acceleration of urban expansion and the development of new residential areas have caused atmospheric NO₂ concentrations to spread toward peripheral areas.

NO₂ concentrations gradually decrease with increasing distance from the city center. Marked decreases in concentration are especially evident toward Akçadağ, Doğanşehir, and Darende. However, this transition does not occur as sharp boundaries, but rather as a continuous spatial gradient. This indicates that atmospheric pollutants are controlled not only by local emission sources, but also by regional transport processes and atmospheric circulation mechanisms. Moderate NO₂ concentrations are notable in Doğanyol, Kale, and Pütürge districts. Although population density is relatively low in these areas, valley topography and fragmented terrain morphology limit atmospheric dispersion. In particular, weakened horizontal air transport in narrow valley systems causes pollutants to accumulate in certain areas. In contrast, Arapgir, Arguvan, Kuluncak, and Hekimhan represent the districts where the lowest NO₂ levels are observed in the study area. In these areas, atmospheric pollutant load remains at lower levels due to low population density, limited transport mobility, and an open topographic structure.

When spatial change is examined by year, NO₂ distribution appears to have shown a relatively balanced structure in 2019 and 2020. However, in 2021 and 2022, high-density zones showed a tendency to expand toward peripheral areas. The expansion observed particularly toward Akçadağ and Doğanşehir is associated with urban growth and increasing transport intensity. In 2024 and 2025, spatial heterogeneity became markedly stronger, and NO₂ levels also increased in semi-urban areas. When topographic factors are considered, the plain and valley systems in the southern parts of Malatya appear to create favorable conditions for pollutant accumulation. Low air circulation, temperature inversions, and stagnant atmospheric conditions cause pollutants to accumulate near the surface in these areas. In contrast, the high-elevation and open topographic areas in the northern parts allow pollutants to disperse more rapidly through stronger wind circulation. This stands out as one of the main environmental mechanisms explaining the low NO₂ levels observed especially around Arguvan and Hekimhan.

To facilitate quantitative comparison among the annual maps, the temporal changes were also evaluated using the available annual descriptive statistics. The mean tropospheric NO₂ value was approximately 18.01 μmol m^−2^ in 2019 and remained similar at 17.97 μmol m^−2^ in 2020. This was followed by an increase to approximately 18.11 μmol m^−2^ in 2021. Although the annual mean remained relatively stable in the following years, the distribution became increasingly characterized by higher localized values. In 2023, for example, the annual mean was approximately 17.87 μmol m^−2^, whereas the maximum reached 40.82 μmol m^−2^. This difference between mean and maximum values became more pronounced toward the end of the study period, with the maximum NO₂ value reaching approximately 46.31 μmol m^−2^ in 2025. These statistics complement the annual maps by providing a quantitative basis for interannual comparison while the year-specific legends preserve the visibility of within-year spatial gradients.

### 3.2 Comparison of NO₂ distribution before and after the earthquake

The comparison between the pre-earthquake period (Pre-EQ), covering 2019–2022, and the post-earthquake period (Post-EQ), covering 2023–2025, shows that the distribution of tropospheric NO₂ across Malatya Province was markedly reorganized in terms of both intensity and spatial organization (Figure 4). Raster-based zonal statistics and period-average distribution maps indicate that the atmospheric NO₂ system became more heterogeneous, more variable, and more fragmented in the post-earthquake period. In the pre-earthquake period, mean NO₂ levels across the province ranged approximately between 19.9 and 21.5 μmol m^−2^. During this period, the distribution of atmospheric pollutants showed a largely regular and continuous spatial pattern. High-concentration zones, particularly concentrated in Battalgazi and Yeşilyurt districts, gradually decreased from the center toward the periphery. While mean NO₂ values in the city center reached approximately 28–32 μmol m^−2^, values in the northern and northwestern districts of Arapgir, Arguvan, Kuluncak, and Hekimhan mostly remained within the range of 15–20 μmol m^−2^. This indicates that emission sources had a relatively stable spatial organization in the pre-earthquake period.

**Figure 4:**
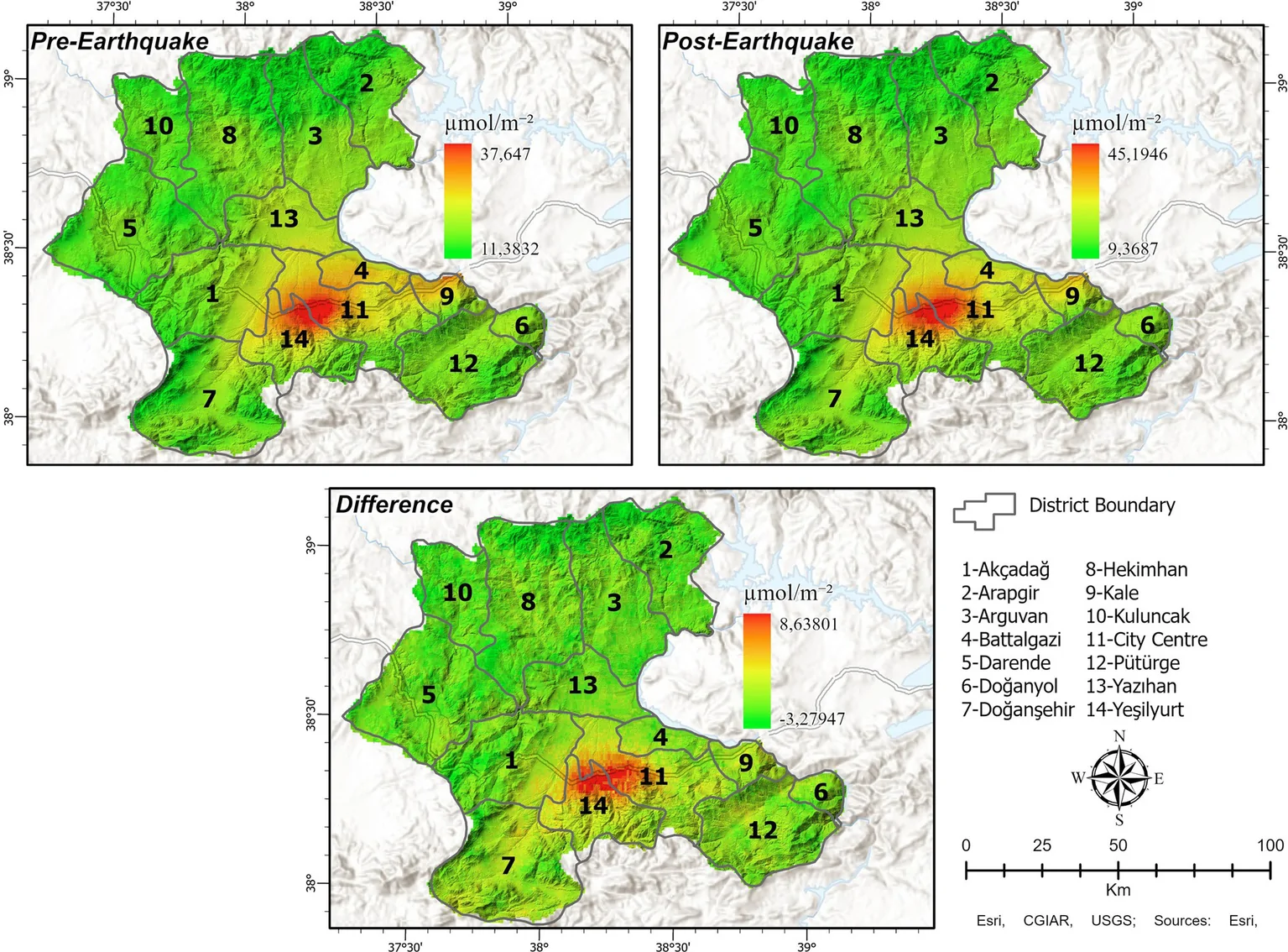
Comparison of pre-earthquake (2019–2022) and post-earthquake (2023–2025) spatial patterns of tropospheric NO_2_ concentrations in Malatya Province.

During the post-earthquake period, the spatial distribution of tropospheric NO₂ became more heterogeneous. The difference surface, calculated as the post-earthquake mean minus the pre-earthquake mean, ranged from −3.28 to +8.64 μmol m^−2^, with a slightly positive spatial mean of +0.21 μmol m^−2^. The largest positive differences were concentrated along the City Centre-Battalgazi-Yeşilyurt corridor, whereas negative or near-zero differences occurred mainly in parts of the northern, northwestern, and peripheral districts. Thus, the results indicate spatially uneven changes, with increases outweighing decreases on average, rather than a uniform province-wide increase. Although the period of increase overlaps with post-earthquake reconstruction, the observed pattern cannot be attributed solely to earthquake-related activities because reconstruction intensity, traffic volume, population mobility, and meteorological conditions were not evaluated directly. The difference map should therefore be interpreted as a descriptive comparison between the two periods.

Topographic factors appear to have become more pronounced in the post-earthquake period. In particular, valley systems and semi-enclosed morphological areas in the southern parts of Malatya create favorable environments for pollutant accumulation due to low air circulation and stagnant atmospheric conditions. In contrast, high-elevation and open topographic areas in the north allow pollutants to disperse more rapidly through stronger atmospheric dispersion processes. Therefore, the low NO₂ levels observed in the northern districts are related not only to low emission intensity, but also to topographic and meteorological conditions.

### 3.3 Mean seasonal variation of tropospheric NO₂ during 2019–2025

The seasonal distribution of tropospheric NO₂ during 2019–2025 shows clear differences among seasons. Multi-year composites derived from Sentinel-5P TROPOMI indicate that these differences occur in both concentration ranges and spatial patterns. Because meteorological variables were not included in the analysis, the observed seasonal variation cannot be attributed to specific atmospheric processes. When seasonal means are examined, the highest province-wide mean tropospheric NO₂ value is observed in winter (21.32 μmol m^−2^), followed by autumn (19.55 μmol m^−2^), summer (18.58 μmol m^−2^), and spring (17.66 μmol m^−2^) (Figure 5). The boxplot shows a similar seasonal ordering in the median values and indicates wider variability in winter and autumn (Figure 6). The seasonal pattern should therefore be interpreted through both central tendency and spatial variability.

**Figure 5:**
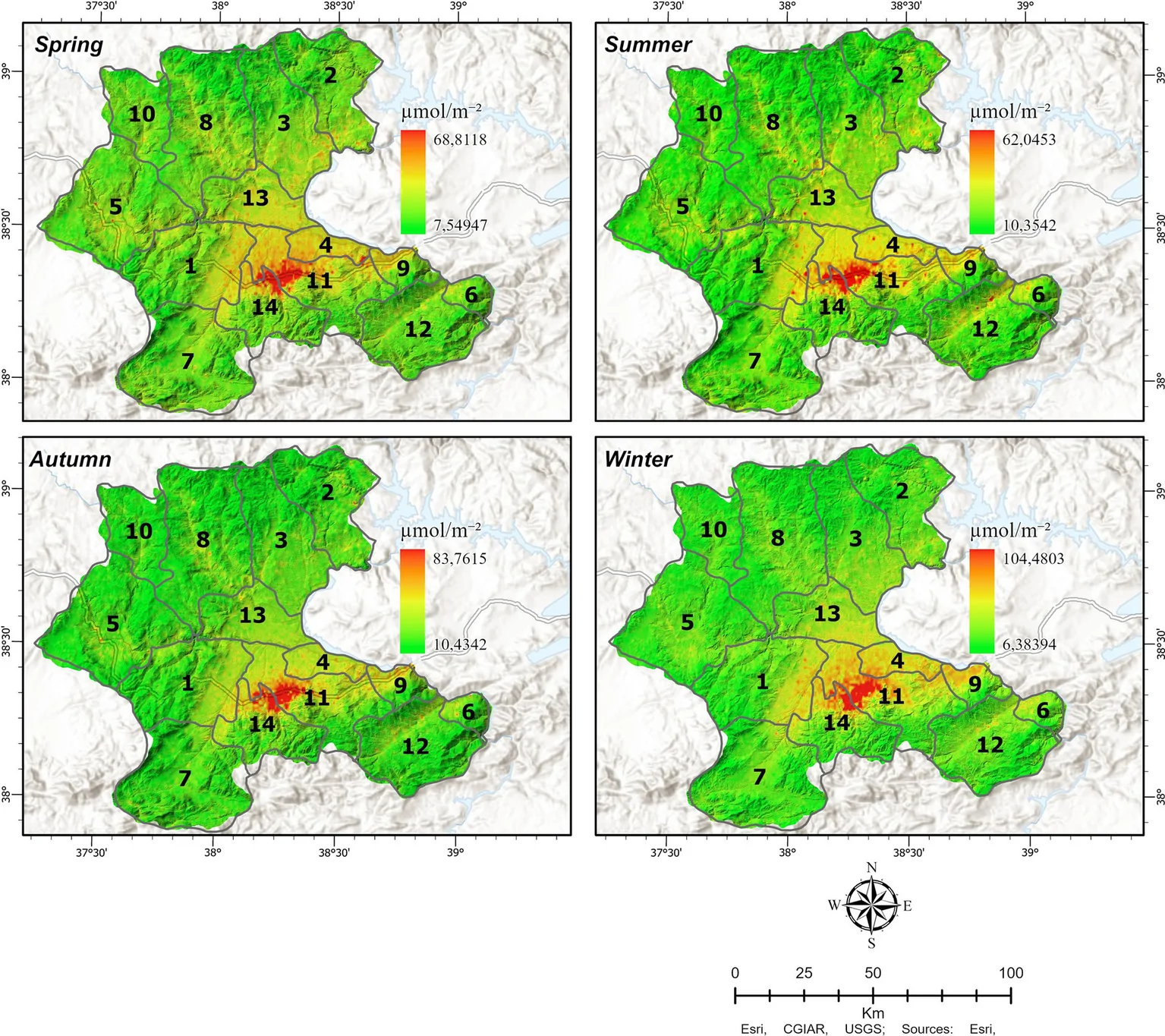
Seasonal distribution of average tropospheric NO_2_ concentrations in Malatya Province for the 2019–2025 period derived from Sentinel-5P TROPOMI observations.

**Figure 6:**
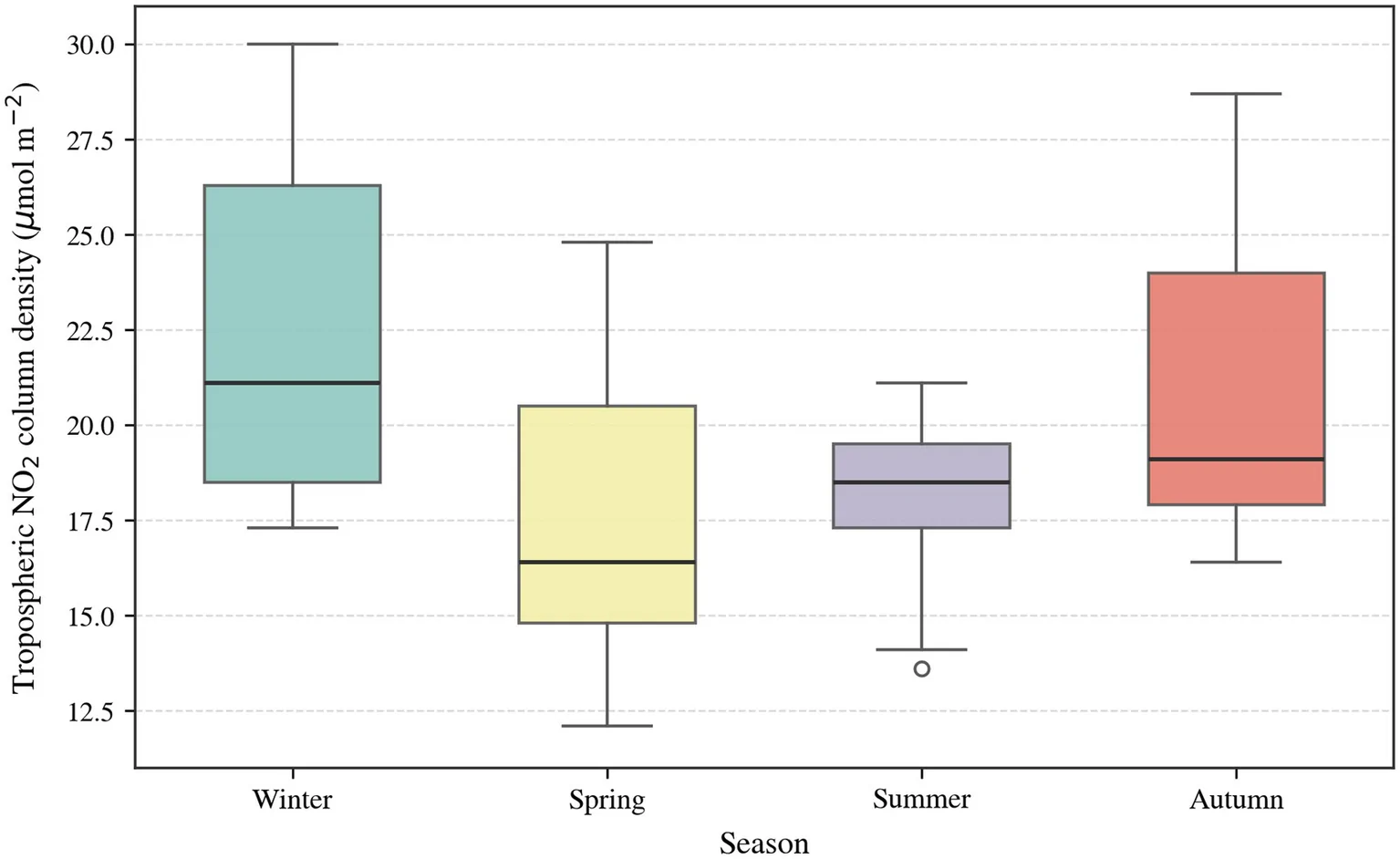
Seasonal distribution of Sentinel-5P-derived tropospheric NO_2_ column density in Malatya Province during 2019–2025. Boxes represent the interquartile range, horizontal lines indicate median values, whiskers show the non-outlier range, and circles denote outliers.

The boxplot analysis presented in Figure 6 shows that the seasonal distribution of tropospheric NO₂ differs markedly in terms of median values, interquartile range, and upper distribution limits. In winter, the median NO₂ value is approximately 21 μmol m^−2^, and the distribution range is wider than in the other seasons. In autumn, the median value is approximately 19 μmol m^−2^, and the upper distribution limit approaches approximately 29 μmol m^−2^. In spring, median NO₂ values are concentrated within the range of approximately 16–17 μmol m^−2^, while the upper distribution limit reaches approximately 25 μmol m^−2^. In summer, median values are approximately 18–19 μmol m^−2^, but the interquartile range and overall distribution width remain more limited. These results indicate that tropospheric NO₂ variability is more pronounced in winter and autumn, while in summer the distribution is concentrated within a relatively narrower range.

When the interquartile range values are examined, the distribution is wider in winter and autumn, whereas spring and summer show more compact distributions. This pattern indicates greater variability in the seasonal NO₂ values represented in the boxplot, particularly during winter and autumn.

Seasonal spatial variability was greatest in Battalgazi and Yeşilyurt, coinciding with the concentration of built-up areas and transport infrastructure. In contrast, the northern districts generally displayed lower and more stable NO₂ patterns. Possible meteorological and anthropogenic explanations for these differences are discussed in Section 4.3.

### 3.4 XGBoost-based NO₂ prediction model and environmental control factors

Annual Sentinel-5P TROPOMI-derived tropospheric NO₂ column density was used as the response variable in the XGBoost model, with elevation, LULC, road density, NDVI, and NDBI as predictors. Applied to the common 1,000 m grid, the model estimated spatial variation in the satellite-derived target and evaluated its nonlinear associations with the selected predictors. The resulting predictions represent modelled TROPOMI column-density patterns rather than ground-level NO₂ concentrations. Taken together, the XGBoost, spatial statistical, and SHAP results indicate that tropospheric NO₂ patterns are associated with several environmental characteristics. The modelled spatial variation reflects contributions from elevation, LULC, road density, NDVI, and NDBI, whose relative importance and direction of association are examined below.

When the environmental variable maps are examined, clear spatial differentiations are observed across Malatya Province (Figure 7). The road density map shows that transport networks are concentrated particularly in Battalgazi and Yeşilyurt districts. The spatial overlap between high road density and tropospheric NO₂ levels in these areas reveals the determining effect of transport-related emissions on atmospheric pollutant load. In contrast, lower NO₂ concentrations are observed together with low road density in the rural areas in the north and northwest. The NDVI distribution shows that vegetation is concentrated particularly in the northern parts and high topographic areas. In contrast, NDVI values are low in densely built-up urban areas. The lower tropospheric NO₂ concentrations observed in areas with high NDVI values support the regulatory effect of vegetation on atmospheric pollutants. Similarly, the NDBI distribution shows that built-up areas are concentrated especially along the Battalgazi-Yeşilyurt axis and that these areas largely overlap with high NO₂ concentrations. This suggests that dense built-up areas increase pollutant accumulation by restricting atmospheric dispersion.

**Figure 7:**
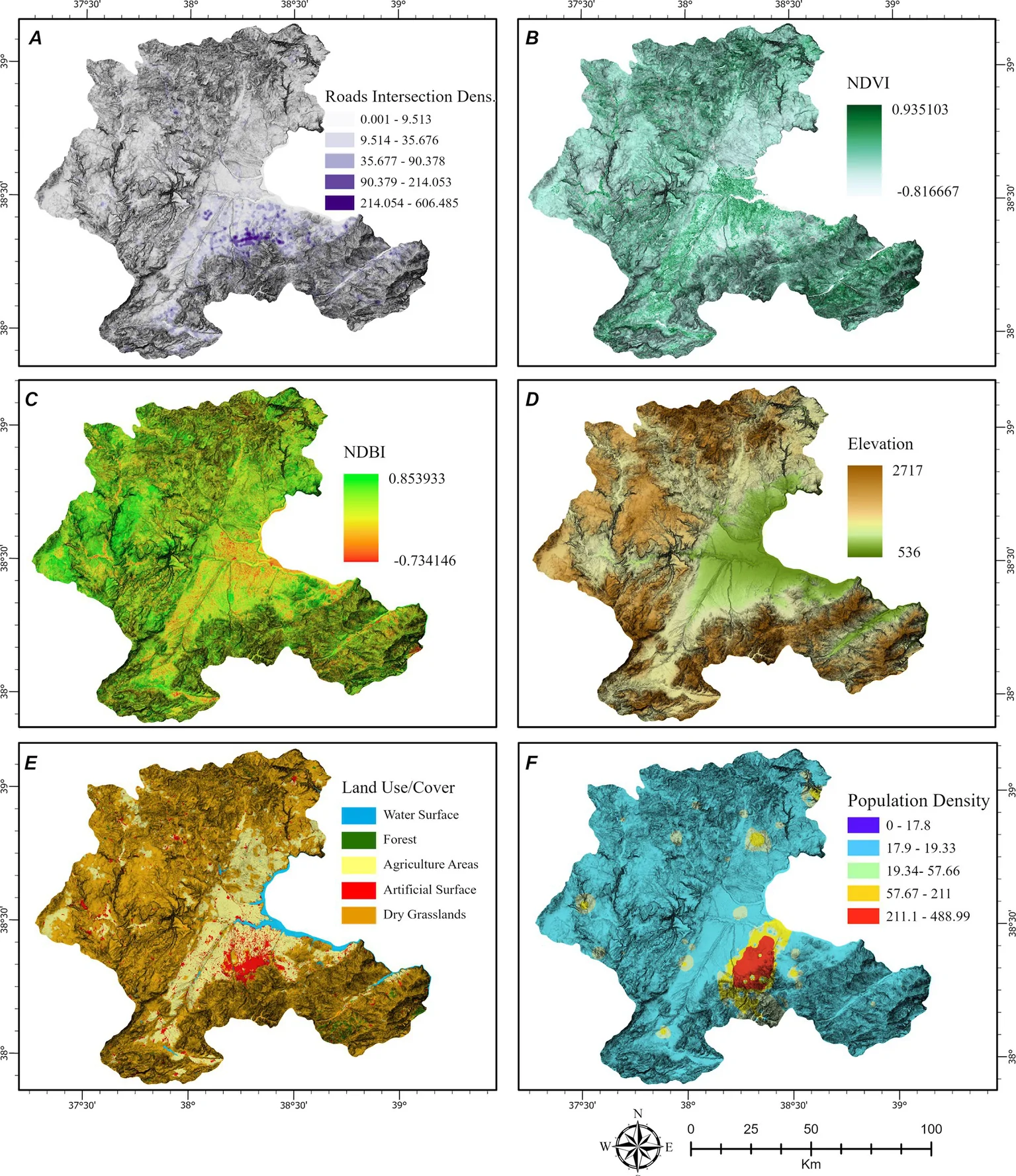
Environmental variables used in the XGBoost model: **(A)** road intersection density; **(B)** Normalized Difference Vegetation Index (NDVI); **(C)** Normalized Difference Built-up Index (NDBI); **(D)** elevation; **(E)** land use/land cover (LULC); and **(F)** population density.

The elevation map shows that topography rises markedly in the northern and southern parts of Malatya, while low-elevation plain and valley systems dominate in the eastern parts. This topographic differentiation has a direct effect on atmospheric dispersion processes. Pollutant accumulation is more intense especially in low-elevation and semi-enclosed morphological areas, while pollutants disperse more rapidly in high topographic areas due to stronger air circulation. When the land use/land cover (LULC) distribution is examined, agricultural areas are seen to cover a large part of the study area, while artificial surfaces are concentrated around the Malatya city center. Tropospheric NO₂ concentrations increase markedly, especially in areas dominated by artificial surfaces. In contrast, lower atmospheric pollutant levels are observed in forested and natural areas. This indicates that different land use classes play a determining role in the tropospheric NO₂ system.

The population density variable was initially included in the model dataset to represent anthropogenic pressure. However, the model outputs show that this variable did not make a meaningful contribution to tropospheric NO₂ predictions. Therefore, road density, land use/land cover, elevation, NDVI, and NDBI variables were taken as the basis in interpreting environmental control factors rather than population density. This indicates that, in the case of Malatya, NO₂ distribution is more strongly associated with the transport network, surface characteristics, topographic structure, and the spatial organization of built-up areas than with population density alone.

When the XGBoost-based prediction map is examined, the model is seen to represent the distribution of tropospheric NO₂ across Malatya Province with high spatial sensitivity (Figure 8). The prediction results show that high NO₂ concentrations are concentrated particularly in Battalgazi and Yeşilyurt districts. In these areas, the NO₂ values predicted by the model are markedly higher than in the surrounding areas and largely overlap with regions characterized by dense built-up areas, transport activities, and high anthropogenic pressure. In Battalgazi district in particular, high-concentration cores occur under the influence of the historical urban center and intensive commercial activities, while in Yeşilyurt district, the recent acceleration of urban expansion and the development of new settlement areas cause atmospheric NO₂ concentrations to spread toward peripheral areas. In contrast, the model predicted lower NO₂ levels in Arapgir, Arguvan, Kuluncak, and Hekimhan districts, located in the north and northwest of the province. This indicates that stronger atmospheric dispersion processes are effective in high topographic areas and that anthropogenic pressure remains at lower levels. Especially in high-elevation open areas, stronger wind circulation causes pollutants to be transported and diluted more rapidly in the atmosphere. These results show that the XGBoost model successfully represents topographic differences and environmental variability.

**Figure 8:**
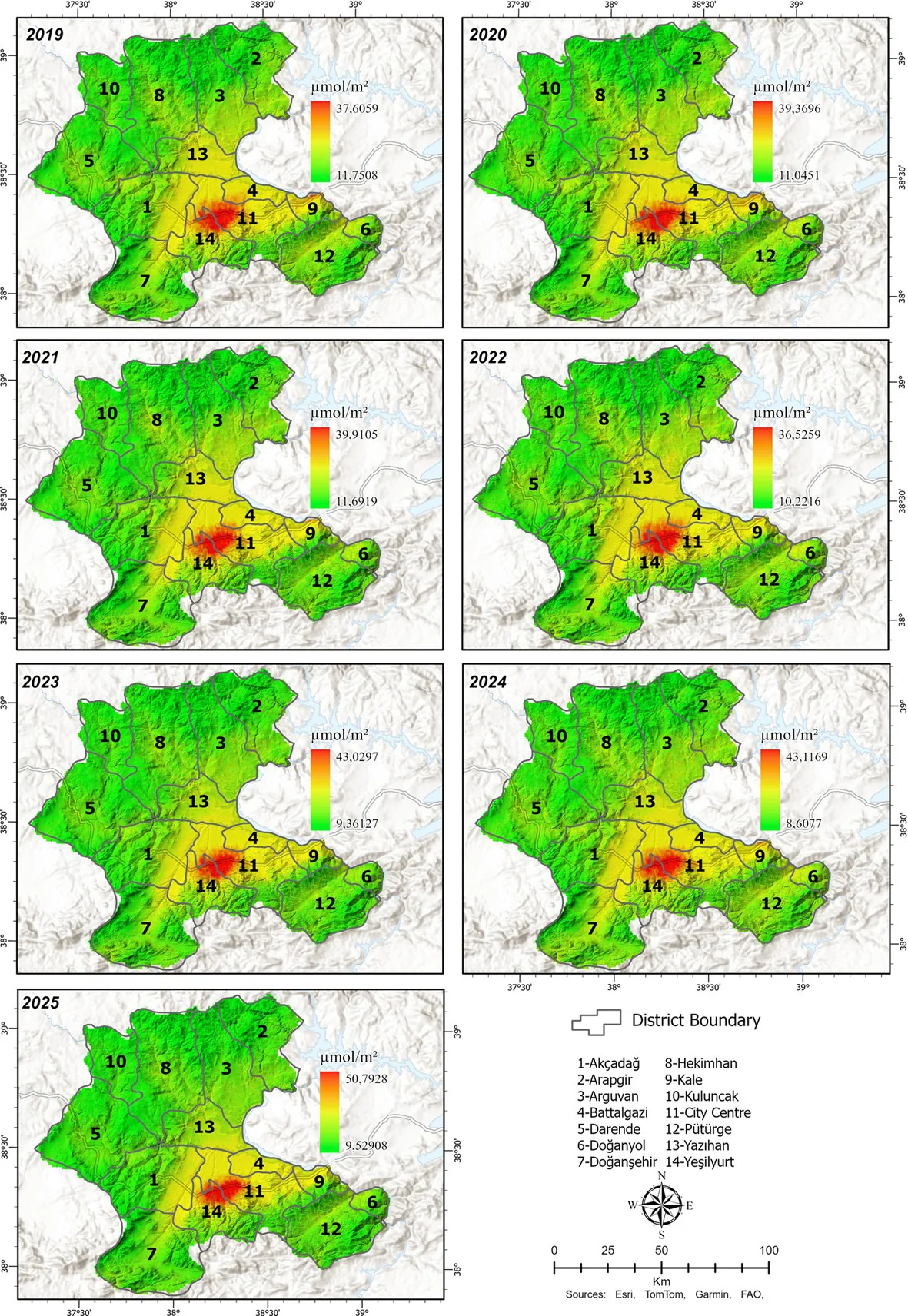
Annual XGBoost-predicted tropospheric NO2 column-density maps for Malatya Province, 2019-2025.

The predicted surface reproduced the main spatial gradients observed in the Sentinel-5P data, particularly the contrast between the Battalgazi-Yeşilyurt corridor and the higher-elevation peripheral areas. The subsequent SHAP analysis was used to quantify the contribution of the selected predictors to this modelled spatial pattern.

To provide a more transparent assessment of model accuracy and generalization capacity, the performance of the XGBoost model was evaluated separately across the training dataset, five-fold cross-validation, and the independent test dataset. On the 80% training dataset, the model achieved an *R*^2^ of 0.7964, an RMSE of 2.016 μmol m^−2^, and an MAE of 1.351 μmol m^−2^. Five-fold cross-validation conducted during model development yielded an *R*^2^ of 0.5978. On the independent 20% test dataset, which was not used during model fitting or hyperparameter optimization, the model achieved an *R*^2^ of 0.5941, an RMSE of 2.816 μmol m^−2^, and an MAE of 1.865 μmol m^−2^. The lower *R*^2^ and higher error values obtained for the independent test dataset relative to the training dataset indicate an expected reduction in predictive performance when the model is applied to unseen observations. Nevertheless, the close agreement between the five-fold cross-validation *R*^2^ (0.5978) and independent test *R*^2^ (0.5941) indicates that out-of-sample performance was consistent across the two evaluation procedures. Overall, these results suggest moderate generalization capacity while also indicating a training–test performance gap that should be considered when interpreting model performance.

The predictive performance of the XGBoost model was evaluated using a scatter plot showing the relationship between observed and predicted NO₂ values (Figure 9). The results indicate that the model generally captured the observed pattern successfully, although the scatter increased at higher NO₂ values. The clustering of points around the 1:1 reference line and the positive trend of the regression line show that the model has a meaningful predictive capacity in explaining the distribution of tropospheric NO₂.

**Figure 9:**
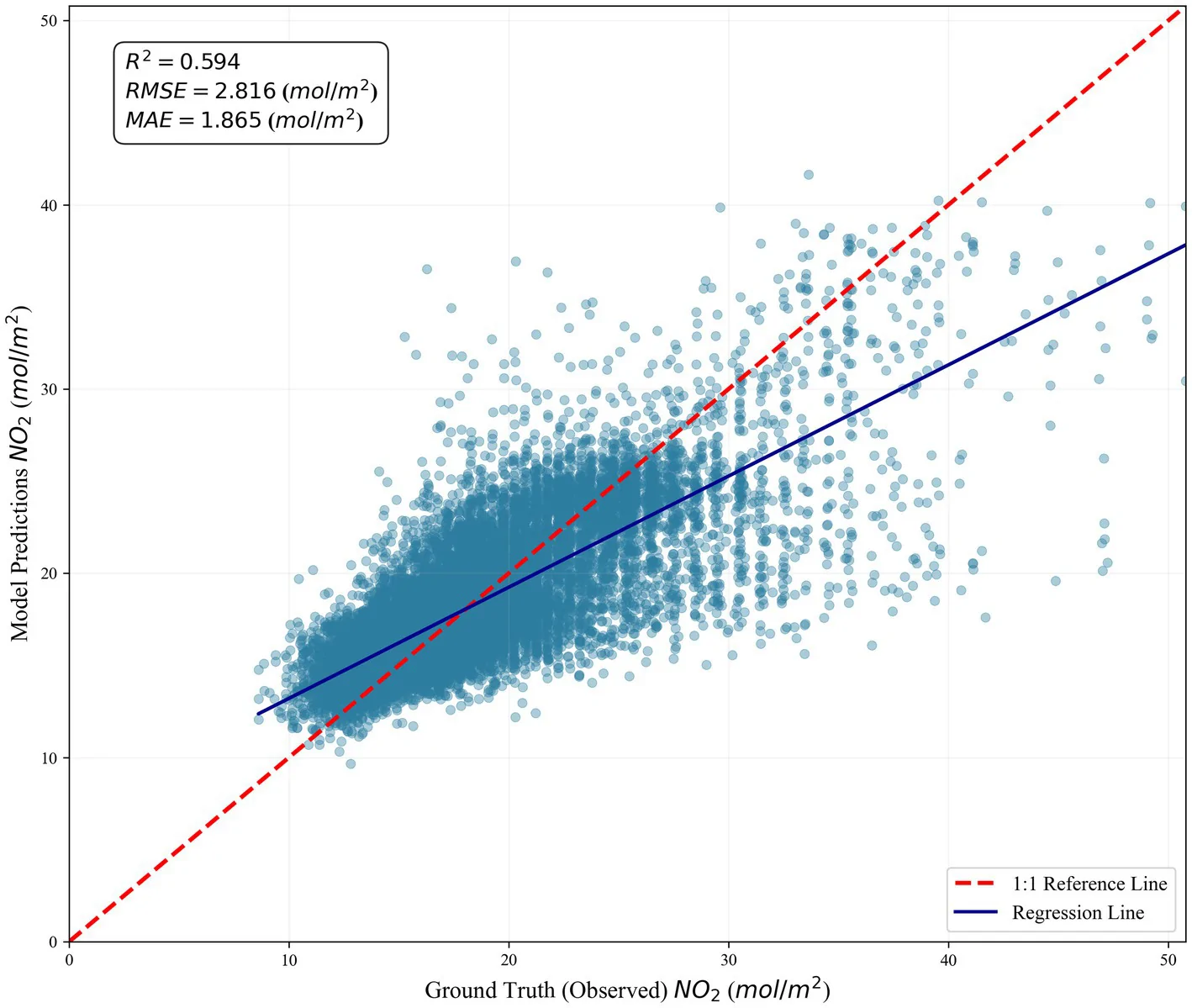
Observed and predicted tropospheric NO_2_ values derived from the XGBoost model.

The annual Global Moran’s *I* analysis revealed consistently strong and statistically significant positive spatial autocorrelation in tropospheric NO₂ throughout the 2019–2025 period (Figure 10). Moran’s *I* values were 0.9911 in 2019, 0.9907 in 2020, 0.9901 in 2021, 0.9893 in 2022, 0.9908 in 2023, 0.9912 in 2024, and 0.9922 in 2025, with all annual statistics being significant at *p* = 0.001. The lowest Moran’s *I* value occurred in 2022 (I = 0.9893), while the highest was recorded in 2025 (I = 0.9922). However, the narrow range of values indicates limited interannual variation in the overall strength of spatial autocorrelation. Tropospheric NO₂ therefore remained strongly clustered throughout the study period.

**Figure 10:**
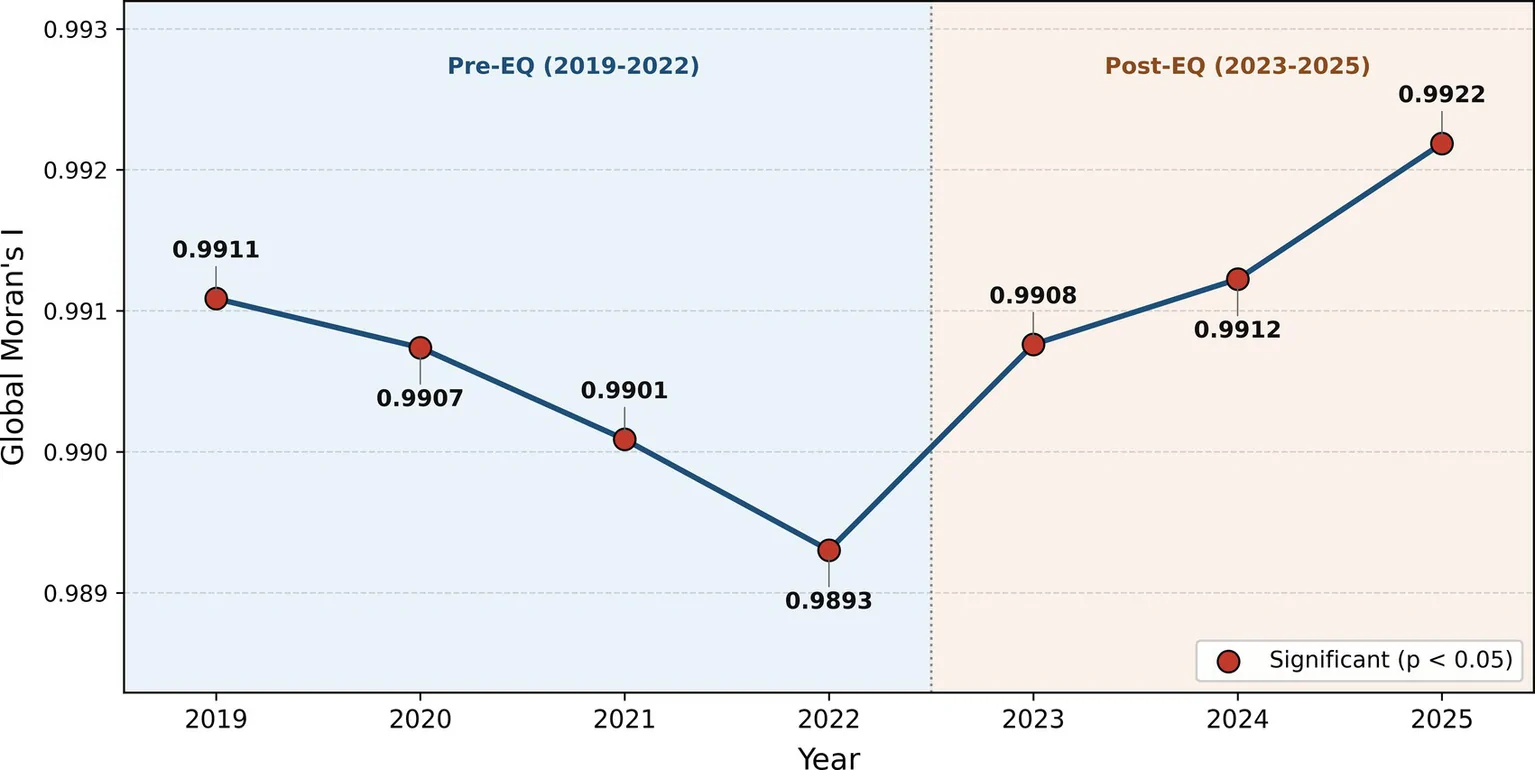
Temporal variation in Global Moran’s *I* for annual Sentinel-5P-derived tropospheric NO_2_ in Malatya during 2019–2025. Spatial weights were defined using Queen contiguity, and statistical significance was assessed using 999 permutations.

Local Moran’s *I* (LISA) analysis further revealed the spatial configuration and temporal evolution of statistically significant NO₂ clusters (Figure 11). High-High (HH) clusters accounted for 17.48, 16.93, 17.90, and 18.21% of valid pixels in 2019, 2020, 2021, and 2022, respectively, compared with 16.31, 17.38, and 16.24% in 2023, 2024, and 2025. Low-Low (LL) clusters represented 31.92, 32.10, 32.31, and 31.15% of valid pixels during 2019–2022 and 32.92, 31.18, and 32.22% during 2023–2025. Although the overall spatial configuration of the principal HH and LL clusters remained relatively persistent, their spatial extent varied among individual years. On average, HH prevalence decreased from 17.63% during the pre-earthquake period (2019–2022) to 16.64% during the post-earthquake period (2023–2025), whereas LL prevalence increased slightly from 31.87 to 32.10%. These values correspond to a 0.99 percentage-point decrease in mean HH coverage and a 0.23 percentage-point increase in mean LL coverage between the two periods. These differences are descriptive and cannot be attributed specifically to the February 2023 earthquakes because emissions, reconstruction activity, meteorological conditions, and other concurrent factors were not controlled in the analysis.

**Figure 11:**
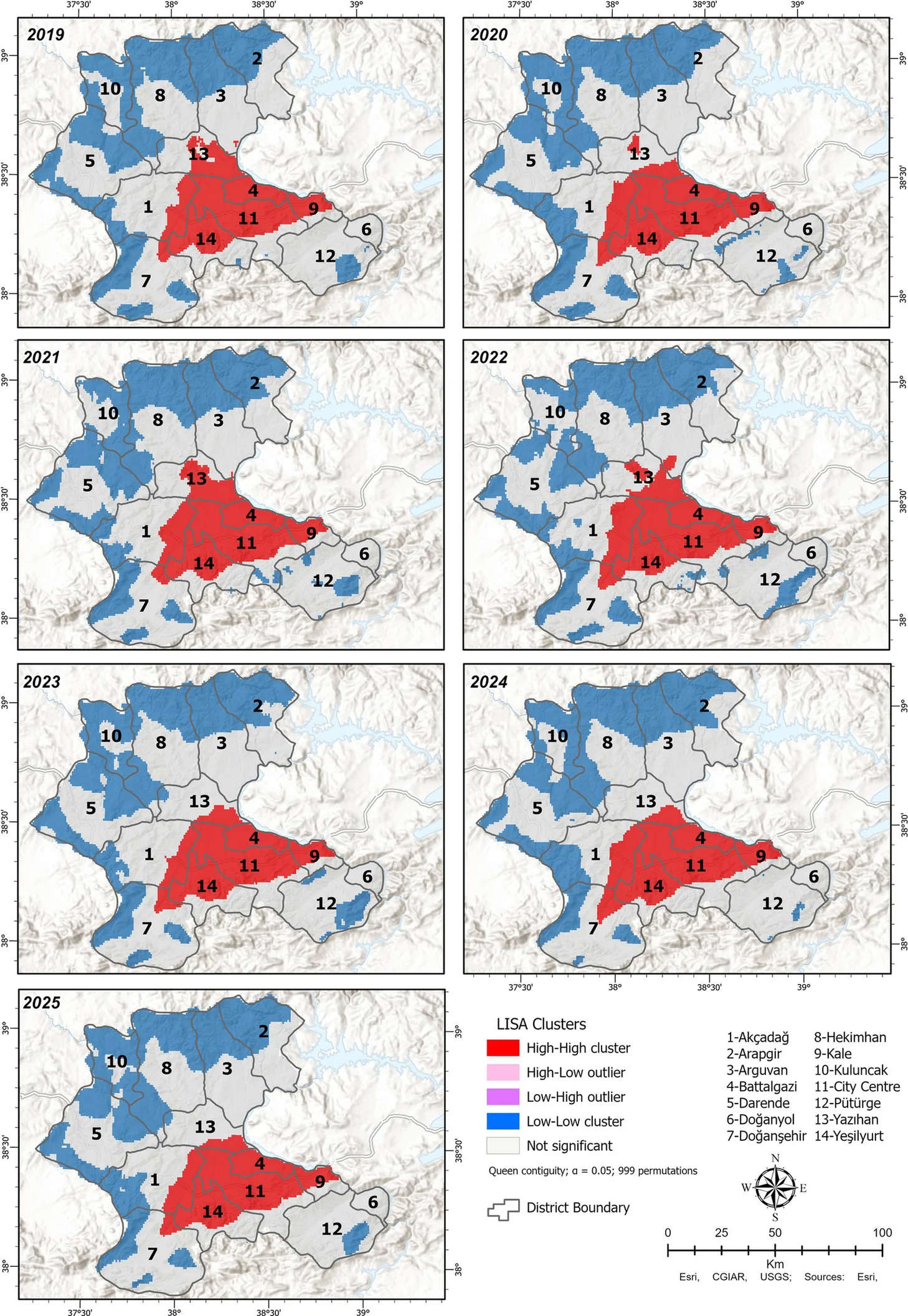
Annual local Moran’s *I* (LISA) cluster maps of Sentinel-5P-derived tropospheric NO_2_ in Malatya during 2019–2025. HH, High–High; LL, Low–Low; HL, High–Low; LH, Low–High; NS, Not significant. Local significance was assessed at *α* = 0.05 using 999 permutations and Queen-contiguity spatial weights.

Temporal anomaly analyses show that tropospheric NO₂ concentrations included marked fluctuations and breaks during the 2019–2025 period (Figure 12). When the monthly mean time series is examined, NO₂ levels show clear seasonal cycles throughout the study period. In particular, NO₂ concentrations increased during summer and autumn, while relatively lower levels were observed in spring and in some winter periods. In the 2020–2021 interval, corresponding to the COVID-19 lockdown period, marked fluctuations and decreases in some periods were observed in NO₂ concentrations. However, in the post-pandemic period, atmospheric NO₂ levels began to increase again. After the February 2023 earthquake, clear anomalies appeared in the time series. In particular, monthly NO₂ averages showed higher variance in the post-earthquake period, and the standard deviation bands widened. This indicates that the atmospheric NO₂ system became more unstable and more heterogeneous in the post-earthquake period.

**Figure 12:**
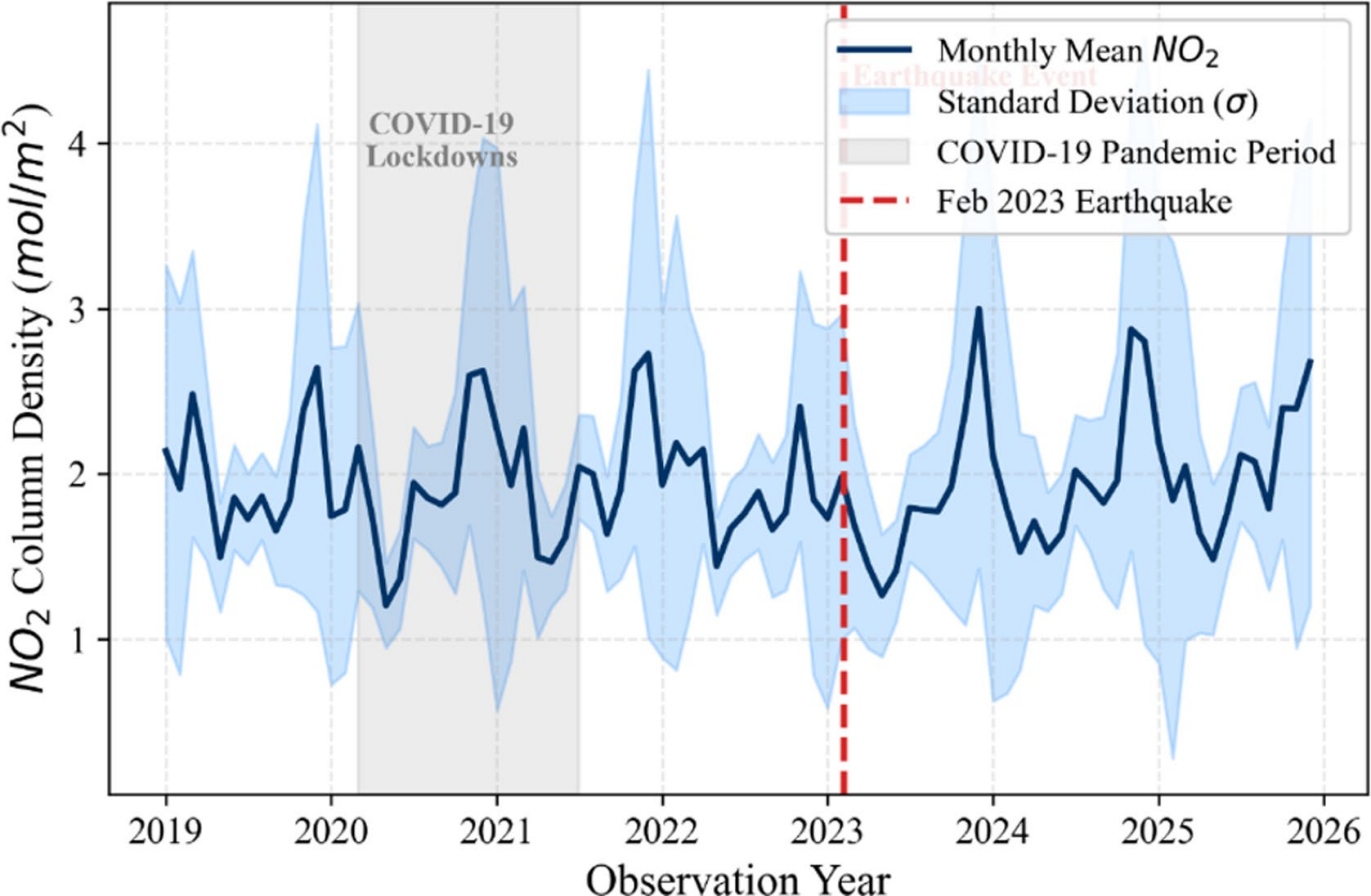
Spatiotemporal trend and anomaly analysis of tropospheric NO2 in Malatya, 2019-2025. The y-axis values are expressed as x 10^-5^ mol m^-2^.

According to the XGBoost variable importance analysis results, Land Use/Land Cover was identified as the variable with the highest contribution to the model (Figure 13). This variable was followed by Elevation and Road Density, respectively. In particular, the fact that the Land Use/Land Cover variable had the highest gain value in the model indicates that tropospheric NO₂ distribution is strongly associated with surface patterns and environmental spatial structure. However, the gain-based importance ranking reflects the contribution of variables to within-tree splits. Since SHAP results evaluate the prediction effect at the pixel level, gain and SHAP outputs were considered together in interpreting variable contributions.

When the XGBoost model, spatial autocorrelation analyses, temporal anomaly assessments, and SHAP interpretations are considered together, it is understood that the tropospheric NO₂ system in Malatya Province exhibits a complex atmospheric structure with a high degree of spatial dependence, temporal variability, and strong control by environmental factors. In particular, the combined effects of topographic structure, land use pattern, transport intensity, and atmospheric processes stand out as the main mechanisms determining the spatial–temporal organization of NO₂ distribution.

**Figure 13:**
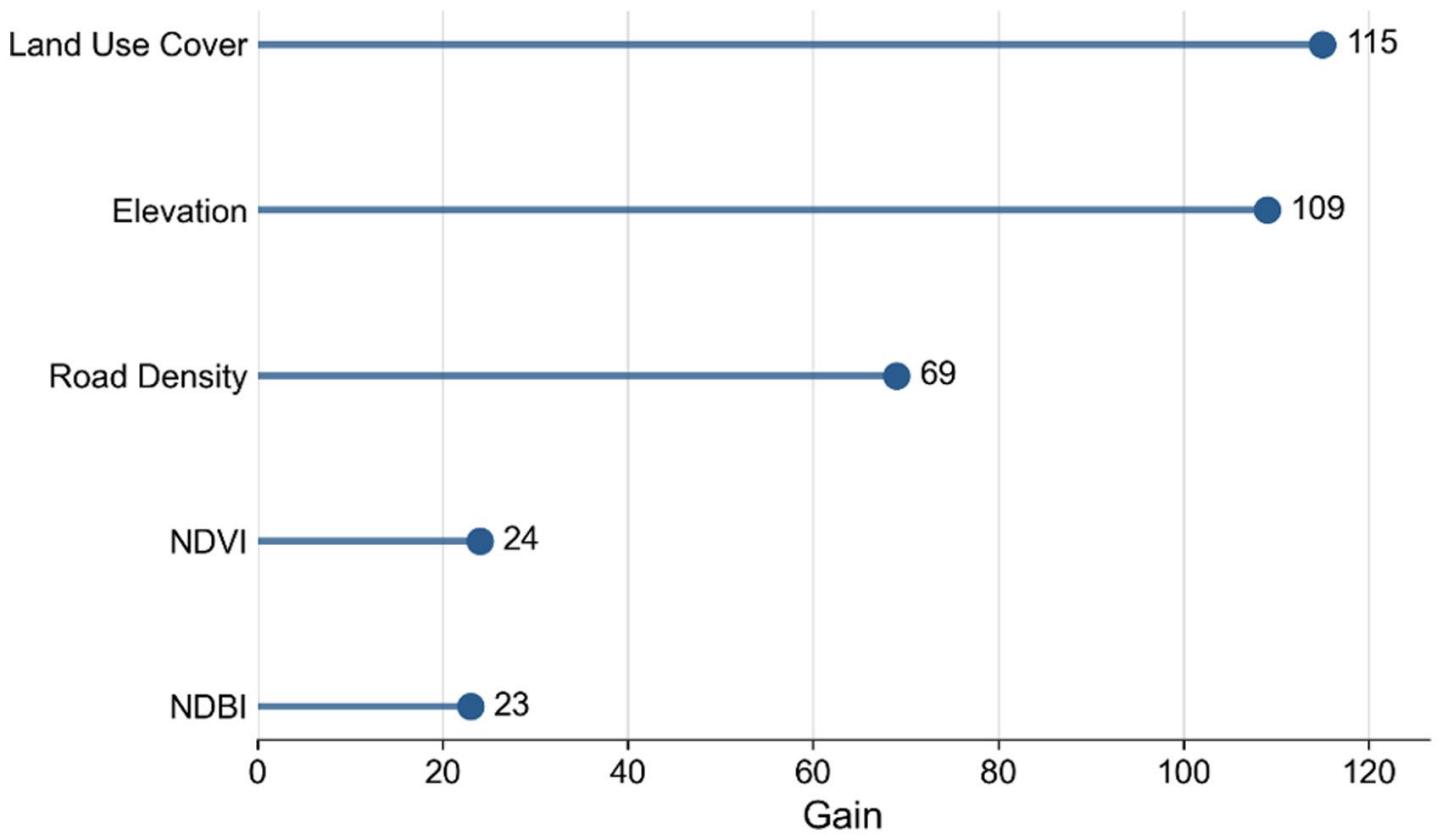
XGBoost feature importance (gain).

The SHAP summary plot clearly shows that the effects of the variables in the model are nonlinear. In particular, low elevation values produce strong positive SHAP effects, while high road density values have a strong increasing effect on the model output. For the NDVI and NDBI variables, the simultaneous occurrence of both positive and negative SHAP values indicates that environmental processes vary depending on the spatial context (Figure 14).

**Figure 14:**
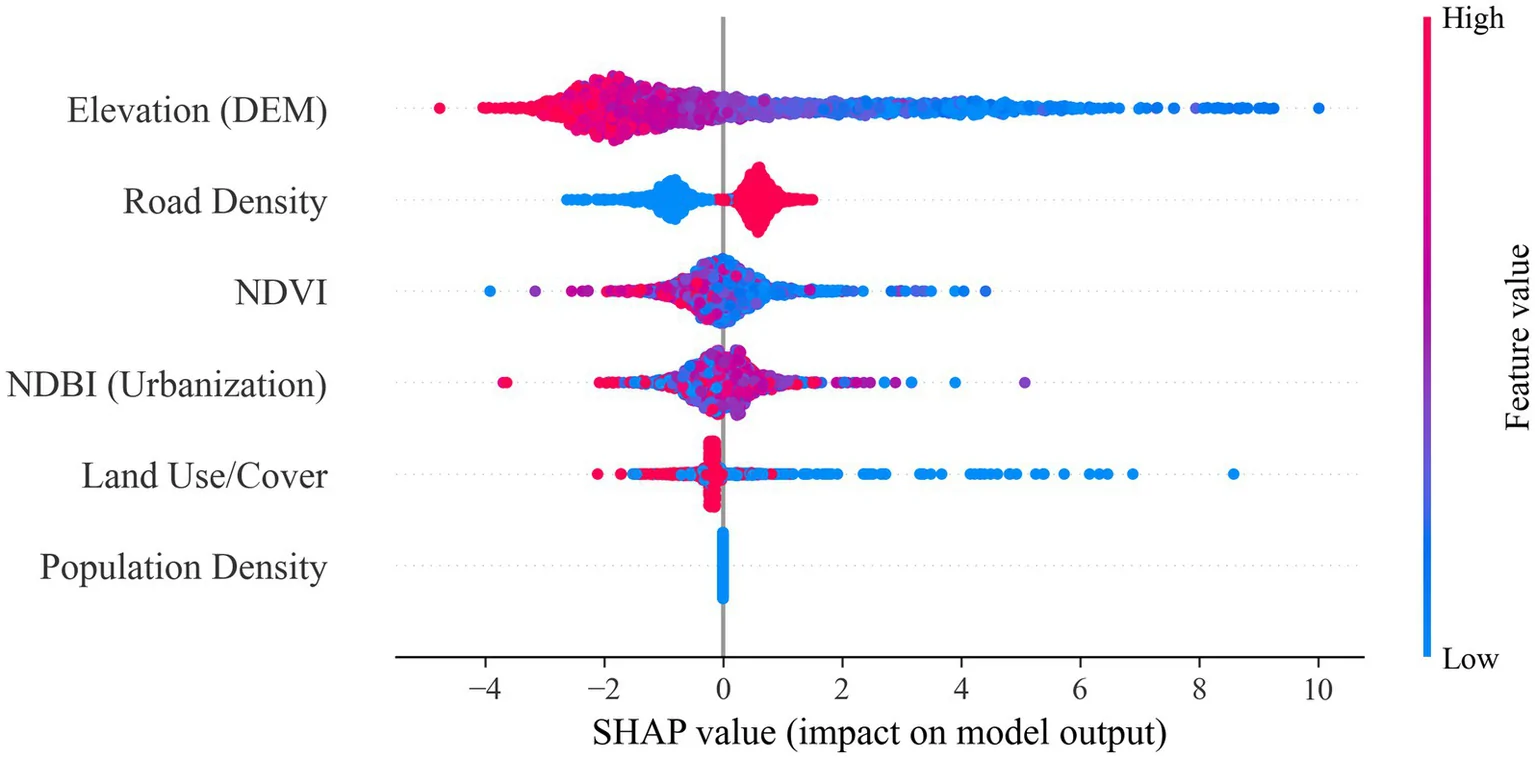
SHAP summary plot. Population density was retained as a predictor but had zero gain and zero SHAP contribution.

### 3.5 Relationship between land use/land cover (LULC) and tropospheric NO₂

The relationship between land use/land cover (LULC) and tropospheric NO₂ concentrations shows that the distribution of atmospheric pollutants is shaped not only by emission intensities, but also by the interaction of surface characteristics, urban morphology, topographic structure, and atmospheric processes. When the XGBoost model, correlation analyses, and SHAP assessments are examined together, LULC components appear to have both direct and indirect effects on the tropospheric NO₂ system. In particular, built-up density, vegetation distribution, and the spatial organization of transport networks stand out as the main environmental factors controlling NO₂ distribution.

The results of the XGBoost variable importance analysis show that the Land Use/Land Cover variable is the parameter with the highest contribution to the model. This indicates that tropospheric NO₂ distribution is largely controlled by surface characteristics and land use patterns. In particular, mean NO₂ values in densely built-up areas in Battalgazi and Yeşilyurt districts were found to be approximately 28–34 μmol m^−2^. In these areas, intense traffic, energy consumption, commercial activities, and the continuity of built-up surfaces are considered the main urban factors that may contribute to increasing the atmospheric pollutant load. In addition, urban surface characteristics such as low permeability and limited air circulation cause pollutants to accumulate in near-surface atmospheric layers.

NDBI analyses support the effect of built-up areas on atmospheric NO₂. SHAP distributions show that high built-up values mostly produce positive SHAP effects. This indicates that dense built-up areas reduce pollutant dispersion by limiting air circulation. NO₂ accumulation is more pronounced especially in semi-enclosed urban morphological areas. In agricultural areas and semi-rural regions, NO₂ concentrations were mostly found to be within the range of 20–26 μmol m^−2^. These areas form transition zones between urban and natural areas and exhibit moderate atmospheric pollutant concentrations. The dominance of open surfaces and the low density of built-up areas allow pollutants to disperse more rapidly. In contrast, tropospheric NO₂ levels in forested and natural areas characterized by high NDVI values remain within approximately 15–22 μmol m^−2^. SHAP distributions for the NDVI variable show that vegetation mostly produces negative SHAP values and therefore has a reducing effect on atmospheric NO₂ concentrations. This indicates that vegetation plays a regulatory role in air quality through both pollutant absorption and lower anthropogenic pressure.

The interaction between topographic structure and LULC forms an important control mechanism on atmospheric NO₂ distribution. In particular, low air circulation and temperature inversion conditions in the densely built-up valley systems in the southern parts of Malatya increase pollutant accumulation. In contrast, pollutant concentrations remain at lower levels in the high-elevation northern parts, where stronger atmospheric dispersion processes are effective. Violin plot analyses conducted among land use classes show that tropospheric NO₂ concentrations differ markedly across LULC classes (Figure 15). In particular, NO₂ concentrations reach the highest levels in the Urban/Industry class, while lower and more homogeneous distributions are observed in the Forest and Pasture classes. These results demonstrate that land use patterns are directly decisive in the spatial organization of the tropospheric NO₂ system.

**Figure 15:**
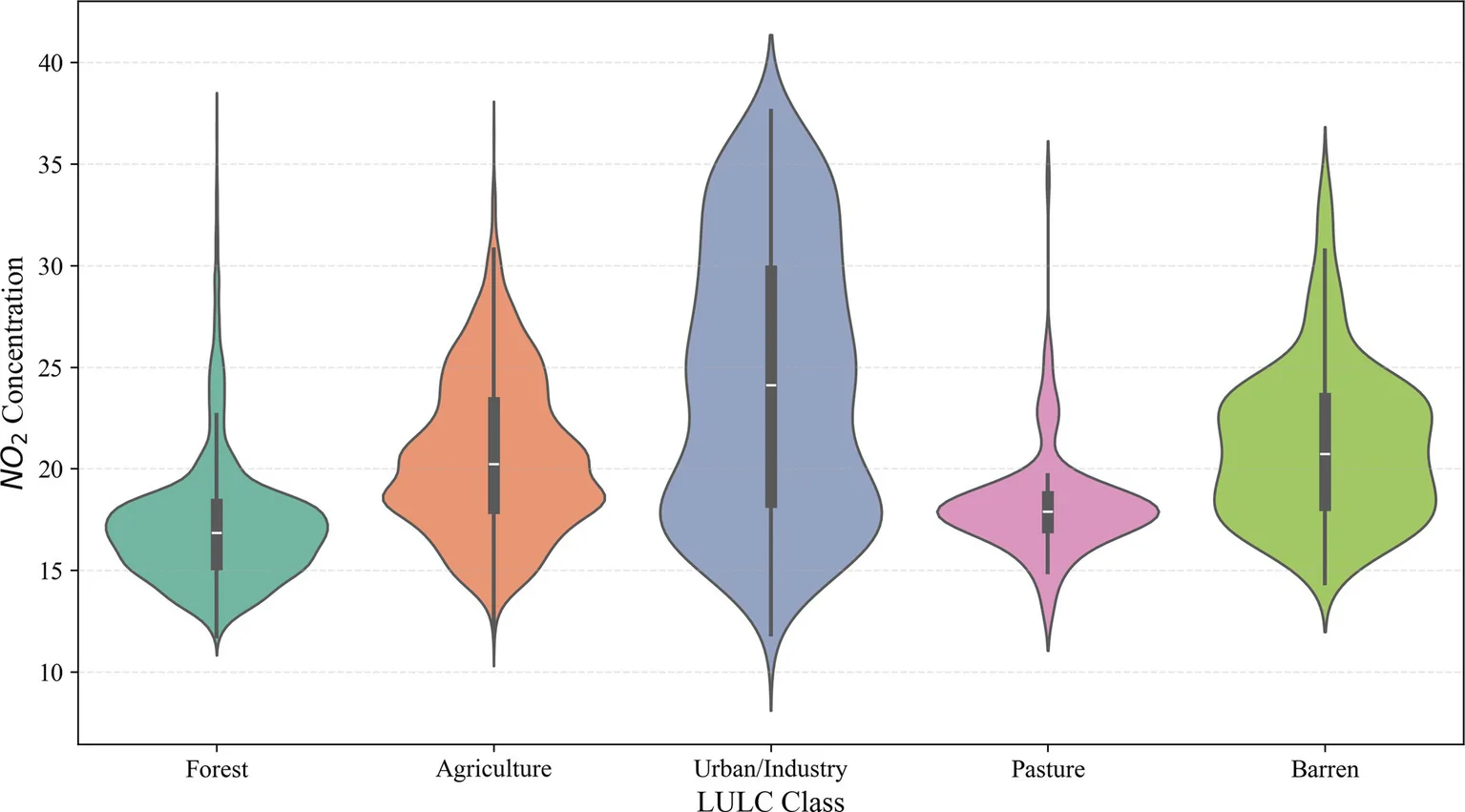
Distribution of tropospheric NO2 across land-use/land-cover classes. Values are expressed in µmol m^-2^.

## 4 Discussion

### 4.1 Land use, topography, and NO₂ exposure

The distribution of tropospheric NO₂ in Malatya does not exhibit a structure that can be explained by a single emission source or by a simple urban center-rural area distinction. The findings indicate a complex spatial pattern in which road density, land use, built-up surfaces, vegetation conditions, and topography operate together. The overlap of high NO₂ concentrations around Battalgazi and Yeşilyurt with the road network and artificial surfaces clearly shows the effect of transport-related emissions and intensive urban functions on the pollutant pattern. This result is consistent with the land use regression and GIS-based air pollution literature, which emphasizes that road networks, land cover, built-up surfaces, and physical geography variables are decisive in explaining the spatial variation of air pollution (9, 11, 12). It is also known that pollutants such as NO₂ and NOx can form sharp spatial gradients around road corridors (48).

The model outputs confirm the strong effect of land use, but also show that this effect should not be interpreted alone. The fact that the Land Use/Cover variable ranks first in the XGBoost gain ranking indicates that land use classes play a strong role in the decision structure of the model. In contrast, the more pronounced contribution of elevation in pixel-level predictions in the SHAP results suggests that the morphological differences between the plains, valleys, and high topographic units of Malatya have an important regulating effect on the NO₂ pattern. These two results do not contradict each other. Gain-based importance reflects the contribution of tree splits within the model, whereas SHAP values reflect the marginal effect of variables on the prediction for each observation (19, 20). Therefore, when LULC, road density, and elevation are evaluated together, it is understood that NO₂ distribution is closely related to both surface characteristics and topographic dispersion conditions.

The role of topography is more evident because of the morphological structure of Malatya. Plain floors, valley corridors, and semi-enclosed morphological areas can create more suitable environments for pollutant accumulation under weak air circulation and atmospheric stagnation conditions. In high-elevation and more open topographic areas, more effective atmospheric dispersion may contribute to lower NO₂ levels. Previous modelling studies have shown that pollutant accumulation in basin and valley systems is related not only to emission intensity, but also to local wind systems and topographic enclosure (15). The NDVI and NDBI results also support this interpretation. The occurrence of low NO₂ patterns in areas with high NDVI values is generally associated with lower anthropogenic pressure, more open morphological conditions, and more limited built-up development. However, the effect of vegetation on air quality is context-dependent. While vegetation can contribute to pollutant retention under some conditions, it may weaken dispersion in dense urban fabrics by limiting airflow (13, 14).

### 4.2 Post-earthquake reconstruction and spatial heterogenization

The comparison of the pre- and post-earthquake periods shows that the NO₂ pattern in Malatya became more heterogeneous and fragmented after 2023. This change does not indicate a homogeneous increase across the province. While increasing trends became more evident in some areas, decreasing trends were observed in the low-density districts in the north and northwest. Therefore, it would not be accurate to interpret the post-earthquake pattern as a unidirectional increase or decrease. A more cautious interpretation is that the reconstruction process, the organization of temporary settlements, changes in transport flows, heavy vehicle mobility, and construction activities may have contributed to local differentiation in NO₂ patterns. Studies showing that debris management, transport mobility, construction activities, and reconstruction pressures can create temporary effects on air quality in post-disaster processes provide a general environmental context for this interpretation (21, 22).

A large part of the post-earthquake air quality literature focuses on particulate matter, dust, and debris-related environmental risks. NO₂, however, is more closely associated with combustion processes, traffic, energy consumption, and heavy vehicle mobility. Therefore, NO₂ changes in Malatya can be evaluated as an environmental exposure signal that may be related to post-earthquake reconstruction, but they should not be interpreted as direct evidence that “the earthquake caused an increase in NO₂.” The strength of this study is that it does not treat the post-disaster process as a stand-alone explanatory factor, but evaluates it together with land use, topography, road density, and temporal variability. This finding is important for post-disaster land use planning. The location of new housing areas, construction logistics, the use of main transport axes, temporary settlement areas, and the redistribution of urban functions may affect long-term environmental exposure patterns. Therefore, post-earthquake planning should consider not only building production and infrastructure repair, but also air quality, transport-related emissions, and exposure concentrations. The case of Malatya shows that monitoring post-disaster spatial reorganization through exposure-related environmental indicators such as NO₂ can provide functional spatial evidence for public-health-sensitive planning.

### 4.3 Seasonal variability and atmospheric context

The seasonal findings show that tropospheric NO₂ dynamics in Malatya cannot be explained by a simple summer- or winter-maximum model. When the map and boxplot results are evaluated together, higher upper distribution limits and wider variability ranges stand out in winter and autumn. In summer, continuous high-density areas along the urban core and southern axis are notable. The observed pattern may reflect the combined influence of meteorological conditions, photochemical processes, seasonal changes in anthropogenic activity, and topographic setting; however, the contribution of these factors could not be quantified within the present model.

The seasonal variation of NO₂ is associated in the literature with emission continuity, atmospheric chemistry, photolysis processes, boundary layer dynamics, and atmospheric lifetime (10, 49, 50). The widening distribution ranges in winter and autumn in Malatya indicate that episodic accumulations may strengthen during periods when dispersion weakens. The spatial continuity observed in summer should not be explained only by photochemical processes. Continuous anthropogenic activities in the city center and along transport axes may also be effective in this pattern. Therefore, in seasonal air quality management, extreme values, distribution ranges, and spatial concentration zones should be evaluated together with seasonal averages.

### 4.4 Methodological contribution of the XGBoost-SHAP approach

The XGBoost model produced a moderate but stable performance in explaining NO₂ distribution in Malatya. The values obtained from the holdout test, $R^{2}=0.594$ , RMSE=2.816 μmol m^−2^, and MAE=1.865 μmol m^−2^, indicate that the model can explain a substantial part of the observed variability, but cannot represent the entire variance. The fact that the best $R^{2}=0.5978\;$ value obtained from cross-validation is close to the holdout result is positive, as it shows that model performance is not overly dependent on a single data split. However, the $R^{2}\;$ value remaining at approximately 0.60 suggests that meteorological variables, traffic volume, industrial sources, fuel consumption, and micro-morphological factors not included in the model also play a role in NO₂ distribution. Therefore, model performance should be evaluated not as “high accuracy,” but as “moderate yet consistent explanatory capacity.”

Methodologically, the added value of the XGBoost framework in this study lies not simply in reproducing the spatial distribution of NO₂ already observable from Sentinel-5P imagery, but in quantifying the multivariate and potentially nonlinear relationships between tropospheric NO₂ and the environmental characteristics of the study area. Conventional GIS-based spatial and temporal analyses are effective for identifying where and when NO₂ patterns change; however, they do not explicitly quantify how multiple environmental predictors jointly contribute to the observed spatial variability. XGBoost complements these analyses by integrating elevation, LULC, road density, NDVI, and NDBI within a single modelling framework capable of representing nonlinear relationships and interactions among predictors (18). The subsequent application of SHAP further enhances the explanatory value of the model by decomposing individual predictions into predictor-specific contributions, thereby allowing the direction and relative magnitude of each variable’s contribution to be examined (20). Accordingly, the principal contribution of the machine-learning component should be understood as the explanatory integration of heterogeneous spatial predictors and the attribution of their relative contributions to Sentinel-5P-derived tropospheric NO₂ variability, rather than as the generation of an independent high-resolution atmospheric observation product. While the gain results show that land use classes have a strong place in the decision structure of the model, the SHAP distribution reveals that elevation plays a more pronounced role in pixel-level predictions. This distinction suggests that the NO₂ pattern in Malatya is shaped by land use information from surface classes together with the dispersion conditions determined by topography. Therefore, the model outputs should not be reduced to a single variable importance ranking, but should be interpreted by reading the gain and SHAP results together (19, 20). Nevertheless, the limitations of the method are clear. Using the LULC variable as a numeric label-encoded predictor may create an artificial ordering effect among classes. In future studies, including LULC classes in the model through one-hot encoding, target encoding, or separate class-based variables may provide more reliable interpretations. The zero variance produced by the population density variable also shows that the variable set representing anthropogenic pressure needs to be redesigned.

### 4.5 Planning implications, limitations, and future research

The findings provide an exposure-oriented perspective on the relationship between land use and public health by evaluating NO₂ as an environmental indicator rather than as a direct health outcome. The relationship between NO₂ exposure and respiratory and cardiovascular health outcomes has been strongly demonstrated in the literature (5–7). However, this study does not use individual health data. Therefore, the findings should be interpreted not as direct epidemiological evidence, but as spatial environmental evidence identifying exposure conditions that are meaningful from a public health perspective. From a planning perspective, the results suggest that areas where high road density, built-up surfaces, and low-elevation morphological units overlap may require closer attention in air quality monitoring and exposure-reduction strategies.

Several limitations should be acknowledged. Although Sentinel-5P TROPOMI observations provide spatially continuous information at the regional scale, they cannot directly capture street-level micro-exposure differences (10, 16, 17). An important limitation of the present study is the absence of independent ground-based NO₂ measurements for validating the satellite-derived and modelled results. Sentinel-5P TROPOMI provides tropospheric NO₂ column density rather than direct measurements of near-surface NO₂ concentration. Consequently, the model outputs should be interpreted as predictions of the spatial variability of Sentinel-5P-derived tropospheric NO₂ columns and not as direct estimates of human exposure to ground-level NO₂. Without collocated ground-monitoring observations, the extent to which variations in tropospheric column NO₂ correspond to near-surface concentrations across Malatya cannot be independently quantified. Future studies should therefore integrate observations from regulatory air-quality monitoring stations, mobile monitoring platforms, or calibrated low-cost sensor networks to enable independent validation and to examine the relationship between satellite-derived column NO₂ and surface-level concentrations. For the same methodological reason, the Sentinel-5P-derived NO₂ values reported in this study were not directly compared with the health-based ambient-air guideline values established by the World Health Organization (5). WHO guideline values for NO₂ are defined as near-surface mass concentrations (μg m^−3^) over specified averaging periods, whereas TROPOMI measures vertically integrated tropospheric NO₂ column density (μmol m^−2^). These quantities represent fundamentally different atmospheric measures and cannot be directly converted or compared without additional information on the vertical distribution of NO₂ and an appropriate column-to-surface conversion or retrieval framework. Therefore, applying WHO concentration thresholds directly to the Sentinel-5P column values could lead to misleading conclusions regarding population exposure or health-related exceedances. Future studies integrating satellite observations with ground-based NO₂ measurements and atmospheric profile information would enable a more appropriate assessment of surface-level concentrations and their relationship with health-based air-quality guidelines. An additional limitation of this study is that meteorological variables were not explicitly included as predictors in the XGBoost model. Atmospheric parameters such as wind speed and direction, air temperature, relative humidity, precipitation, boundary-layer height, and atmospheric stability can substantially influence the transport, dispersion, chemical transformation, and accumulation of NO₂. Consequently, part of the unexplained spatial and temporal variability in the model may reflect meteorological processes that are not represented in the current predictor set. This limitation is particularly relevant when interpreting seasonal differences and short-term anomalies, because variations in atmospheric dispersion conditions may modify NO₂ concentrations independently of land use, road density, vegetation, or topography. Therefore, the relationships identified in this study should be interpreted as associations between NO₂ patterns and the included spatial-environmental predictors rather than as a complete representation of all processes controlling atmospheric NO₂. Future studies should integrate meteorological reanalysis or station-based variables, including wind speed, temperature, relative humidity, precipitation, and boundary-layer characteristics, into the modelling framework to improve explanatory performance and strengthen the interpretation of temporal variability. Future studies should integrate ground-based NO₂ measurements, mobile sensor observations, meteorological reanalysis data, traffic flows, reconstruction records, and health-related indicators. Methodologically, spatial cross-validation and alternative LULC encoding strategies would further improve model generalizability and interpretability.

## 5 Conclusion

This study assessed the temporal, seasonal, and spatial patterns of tropospheric NO₂ in Malatya Province during the 2019–2025 period by integrating Sentinel-5P TROPOMI observations with GIS-based environmental variables, XGBoost modelling, SHAP interpretation, and spatial autocorrelation analysis. The findings show that NO₂ distribution in Malatya cannot be explained by emission intensity alone. Instead, it is shaped by the combined influence of land use/land cover, road density, built-up surfaces, vegetation conditions, elevation, and topographic dispersion processes.

The spatial results indicate that high NO₂ concentrations are concentrated mainly along the Battalgazi-Yeşilyurt urban axis, where dense transport networks, artificial surfaces, and intensive urban functions overlap. Lower NO₂ levels are observed in higher-elevation and lower-density areas, particularly in the northern and northwestern parts of the province. The model results support this spatial pattern and show that the XGBoost-SHAP framework provides a moderate but consistent and interpretable basis for explaining the environmental controls of tropospheric NO₂. Importantly, the XGBoost outputs represent modelled variability in Sentinel-5P-derived tropospheric NO₂ column density and should not be interpreted as independent ground-level NO₂ estimates or as genuinely higher-resolution atmospheric observations. The spatial autocorrelation analysis further confirms that NO₂ concentrations exhibit a clustered structure rather than a random spatial pattern.

The comparison between the pre-earthquake period of 2019–2022 and the post-earthquake period of 2023–2025 shows that NO₂ patterns became more fragmented and spatially heterogeneous after the earthquakes. This pattern should not be interpreted as a uniform increase across the province. Rather, the findings suggest that reconstruction activities, changes in transport flows, temporary settlement organization, heavy vehicle mobility, and local spatial reorganization may have contributed to the differentiation of exposure conditions. In this respect, the study provides interpretable, exposure-oriented spatial evidence for public-health-sensitive land use planning and post-disaster urban management.

## Data Availability

The raw data supporting the conclusions of this article will be made available by the authors, without undue reservation.
